# Dysbiosis and Its Discontents

**DOI:** 10.1128/mBio.01492-17

**Published:** 2017-10-10

**Authors:** Katarzyna B. Hooks, Maureen A. O’Malley

**Affiliations:** a Centre de Bioinformatique de Bordeaux, Centre de Génomique Fonctionnelle de Bordeaux, University of Bordeaux, Bordeaux, France; b Immunoconcept, CNRS UMR 5164, University of Bordeaux, Bordeaux, France; University of British Columbia

**Keywords:** dysbiosis, homeostasis, microbial diversity, microbiome, microbiota

## Abstract

Dysbiosis is a key term in human microbiome research, especially when microbiome patterns are associated with disease states. Although some questions have been raised about how this term is applied, its use continues undiminished in the literature. We investigate the ways in which microbiome researchers discuss dysbiosis and then assess the impact of different concepts of dysbiosis on microbiome research. After an overview of the term’s historical roots, we conduct quantitative and qualitative analyses of a large selection of contemporary dysbiosis statements. We categorize both short definitions and longer conceptual statements about dysbiosis. Further analysis allows us to identify the problematic implications of how dysbiosis is used, particularly with regard to causal hypotheses and normal-abnormal distinctions. We suggest that researchers should reflect carefully on the ways in which they discuss dysbiosis, in order for the field to continue to develop greater predictive scope and explanatory depth.

## PERSPECTIVE

As microbiome research has flourished, so has the use of the term “dysbiosis.” Particularly when patterns in microbiota are linked to human health and disease states, dysbiosis is often invoked as a state that mediates these associations. Although a few commentators have noted the looseness of what dysbiosis means and is doing scientifically (e.g., references 1 and 2), its use shows no sign of declining. We sought to understand more exactly how microbiome researchers apply this term and for what purposes. More specifically, we wanted to find out whether dysbiosis is helping or harming microbiome research. To do this, we first uncover the term's historical roots. We then carry out an analysis of contemporary dysbiosis statements in order to understand how frequently the term appears and in which senses. We examine both broad uses and some specific examples to identify a series of problems. We argue that these problems may affect how microbiota research develops from an association-focused science into a more fine-grained explanatory science.

## EARLY HISTORICAL BACKGROUND

**FIG 1:**
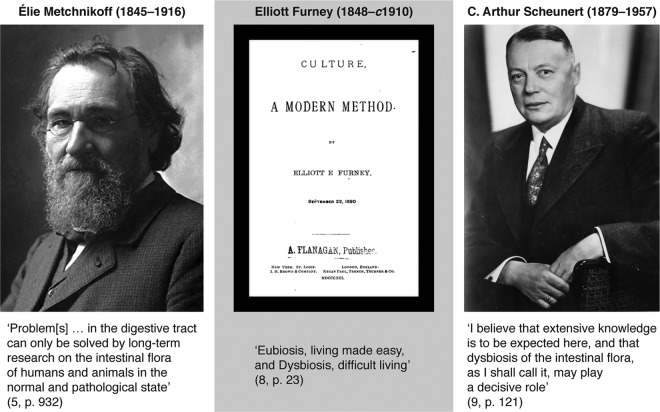
The contributions of Metchnikoff, Furney, and Scheunert to dysbiosis thinking. Metchnikoff photo from Les Prix Nobels 1908; Furney book cover from https://babel.hathitrust.org/cgi/pt?id=nnc1.1000947504;view=1up;seq=5; and Scheunert photo from http://www.ullmann-bernau.de/Ahnengemeinschaften (used with permission of the creator of the digital rendition, Steffen Ullmann [original photographer unknown]).

Dysbiosis has a long history that begins with the first analyses of the human gut “microflora” in the late 19th and early 20th centuries. Although some contemporary sources say Élie Metchnikoff (Fig. 1) was the source for today’s use of dysbiosis (e.g., references 3 and 4), this is not directly the case. Metchnikoff, the Nobel Prize-winning zoologist-immunologist and longevity researcher, never mentioned the word dysbiosis. However, he did call attention to resident microorganisms and their different effects on the human body, which he thought could be “normal” or “pathological” (5). Metchnikoff was very much concerned that humans had inherited a large intestine that was of little use to them. It functioned “merely” to provide conditions favorable to bacteria, many of which he thought could not possibly be helpful and were probably shortening human life (6, 7). He imagined that one day surgery would allow the routine surgical removal of everyone’s “useless” large intestine. In the meantime, yogurt bacteria and the avoidance of raw food would suppress the flourishing of harmful “putrefying” bacteria.

A physician-novelist of the same era, Elliott Furney (Fig. 1), used both “eubiosis” and “dysbiosis” in his science fiction account of animal cloning and regeneration (8). However, he deployed these terms in a very different sense than is relevant for microbiology. Furney was advocating a form of positive eugenics and was also enthusiastic about the idea of growing chimera organisms as servants (e.g., chimpanzee and human cell mixtures). His rather far-fetched view of culturing was that cloned cells had the memories and learned capabilities of the original organism. Citing him as the source of the dysbiosis concept is thus misleading.

It took until the German medical and veterinary literature of the early 20th century to find the first discussions of dysbiosis that resonate with today’s conception, usually in the phrase “Dysbiose der Darmflora” (the latter meaning gut “flora,” which was the terminology of the time). The first microbiological use of this term appears in C. Arthur Scheunert’s 1920 paper on the relationship between intestinal “flora” and bone inflammation in horses (9) (Fig. 1). He claimed that gut dysbiosis was implicated in equine disease and that it could be prevented by more hygienic stables and water. Scheunert suggested that he had coined the word “dysbiosis” himself: “Ich glaube, daß hier weitgehende Erkenntnisse zu erwarten sind und jener Dysbiose der Darmflora, **wie ich sie bezeichnen möchte,** dabei vielleicht eine entscheidende Rolle zugeschrieben werden muß” (translation: “I believe that extensive knowledge is to be expected here, and that dysbiosis of the intestinal flora, **as I shall call it**, may play a decisive role”) (emphasis added) (9). Later in his career, Scheunert became involved in digestion research in humans, especially prison inmates during World War II, but the main legacy of this project is strong ethical criticism (10).

We suggest that although these three authors provide very different resources for thinking about dysbiosis, their contributions nevertheless ended up being combined historically: Metchnikoff urged scientists to think about normal and abnormal microorganismal effects on hosts, Furney promoted the words dysbiosis and eubiosis but gave them a rather dubious fictional context, and Scheunert brought together the terminology and relevant microbe-host research (Fig. 1).

## THE HISTORICAL BRIDGE: HELMUT HAENEL

**FIG 2:**
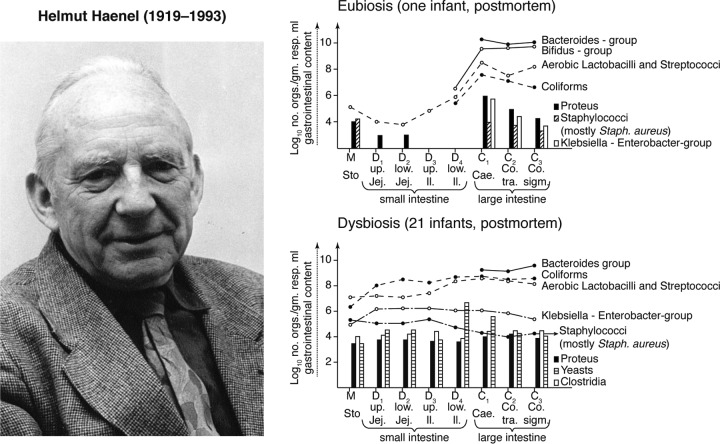
Helmut Haenel’s representations of dysbiosis and eubiosis. Image of Haenel used with permission of the Berlin-Brandenburgische Akademie der Wissenschaften. Figures of eubiosis and dysbiosis reconstructed from the work of Haenel (Fig. 4 and 5 in reference 16).

Regardless of the precise details of the older history, the revival of dysbiosis in scientific literature in English occurred via the prolific work of Helmut Haenel, a “microecologist” of the postwar era in Potsdam, Germany (see reference 11 for historical context). Haenel made repeated mention of dysbiosis and gave it its contemporary gloss of change and imbalance, which could be contrasted to the positive “normal” state that he called eubiosis (12) (Fig. 2). He cites Scheunert, who was the director of the Institute for Nutrition at Potsdam, as the source of his dysbiosis concept. A number of other contemporaneous Eastern European researchers also elaborated on dysbiotic or “dysbacteriotic” states of intestines (e.g., 13 and 14).

Haenel carried out extensive analyses of human intestinal contents and feces in order to characterize the microorganisms in the human gut at a range of ages. He found that convalescing children, although deemed “clinically healthy,” possessed “a faecal microbiocenosis [microbiota] … so altered that it should not be understood as eubiosis with normal composition but as dysbiosis with disturbed relations” (15). Haenel then proposed criteria for detecting these disturbed relations by quantifying variations from typical bacterial counts for the intestines and feces (16) (Fig. 2). Eubiosis was the inverse condition of a “normal, naturally occurring, reproducibly composed, microbiocenosis of a healthy organ” (12). Near the end of his working life, Haenel became concerned about how much was truly known about dysbiosis and particularly the more popular positive word “homeostasis” (17). His suspicions about the beneficial state may have arisen because, like Metchnikoff, he thought that the “intestinal microflora has no general important benefit” (12) and existed merely as an evolutionary consequence of selection “to reduce the frequency of emptying the bowel” (16).

Haenel acknowledged that culturing could not reveal the total composition of the microbiota (e.g., 12) but thought that his organismal counts worked as effective indicators of the global “microflora” state. He also cautioned against beliefs that results achieved with gnotobiotic animal models could be translated straightforwardly into human biology (18). His research raised questions about host-microbiota stability, imbalance, and responses to perturbations, such as the effects of diet (including breast milk), caesarean sections, and antimicrobial treatments on intestinal microbe communities (e.g., 17 and 18). These same concerns, along with the language of dysbiosis (and, somewhat less frequently, eubiosis), have made their way into today’s contemporary literature on the microbiome—itself a term with a much older legacy than usually acknowledged (19).

A few explicit attributions to Haenel are made in some of today’s microbiome literature (plus, more misleadingly, to Furney) for the word dysbiosis. However, very little of this recent literature tries to be more elaborate or precise when discussing dysbiosis than Haenel and other 1970s writers were. Despite this, the term seems more concrete now than it has ever been, and it is not clear that this is justified. To find out more about contemporary uses of dysbiosis, we systematically investigated today’s microbiome literature.

## CONTEMPORARY DEFINITIONS OF DYSBIOSIS

Our analysis focused on over 9,000 PubMed abstracts in which the MeSH term “microbiota” appears (this term also captures “microbiome” [see Text S1 in the supplemental material]). Around 5% of these articles use the term “dysbiosis.” Although microbiota articles span both medical and environmental journals (Text S1), dysbiosis is discussed almost exclusively in the context of human health (or animal models of human health). The main medical contexts for dysbiosis are inflammatory bowel disease, *Clostridium difficile* infections, and autoimmune disorders.

10.1128/mBio.01492-17.1 TEXT S1  1. Outline of methodology. 2. Dysbiosis indices. Download TEXT S1, DOCX file, 0.1 MB. Copyright © 2017 Hooks and O’Malley. 2017 Hooks and O’Malley This content is distributed under the terms of the Creative Commons Attribution 4.0 International license.

### Semi-automated analysis of all uses of dysbiosis.

**FIG 3:**
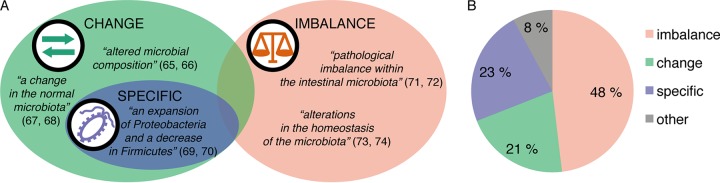
Definitions of dysbiosis. (A) Types of definitions with sample quotes, sometimes slightly paraphrased. References: 65, Maynard et al. (2012); 66, Arrieta et al. (2016); 67, Nibali et al. (2014); 68, Williams and Gallo (2015); 69, Lewis et al. (2015); 70, Youmans et al. (2015); 71, Lawley et al. (2012); 72, Jones et al. (2014); 73, Bested et al. (2013); 74, Sommer and Bäckhed (2013). (B) Popularity of different definitions.

After identifying 554 dysbiosis sources, we categorized their explicit and semi-explicit definitions of dysbiosis into three categories. Similarly to the work of Olesen and Alm (1), we found three main uses of dysbiosis: as general change in the microbiota composition (e.g., alteration, perturbation, abnormal composition, and loss of diversity), as an imbalance in composition (almost always deemed to have negative effects), and as changes to specific lineages in that composition (any named taxon change). Conceptually, we represent these categories as partly overlapping but differing in emphasis (Fig. 3A).

“Imbalance” is the most common definition, in almost half of the total abstracts. Details of the nature of this “imbalance” are only sometimes stated. It mostly refers to broad compositional change, but sometimes more specific adjustments to ratios of organisms are invoked. More than one in five of the definitions belong to the general “change” category, which includes very broad observations of “alteration” as well as specifications that these changes are associated with disease. The narrowest definition, “specific,” occurs in almost a quarter of abstracts. As long as the changes were named taxonomically, we included them in this category, whereas unspecified compositional or functional increases and decreases we left in “change.” Specific changes refer to taxon shifts that may be as fine-grained as increases in different strains of *Escherichia coli* or as encompassing as changed proportions of its phylum of *Proteobacteria*. Increases at the genus level are also mentioned (e.g., *Staphylococcus*, including methicillin-resistant *S. aureus* [MRSA]), along with families, orders, and classes. Finally, about 1 in 12 definitions did not fit our categorization scheme (Fig. 3B), because they either invoked multiple aspects of dysbiosis or made atypical statements (see Table S1 in the supplemental material). These we labeled “other” and will not analyze further.

10.1128/mBio.01492-17.2 TABLE S1  List of short definitions. Download TABLE S1, PDF file, 0.1 MB. Copyright © 2017 Hooks and O’Malley. 2017 Hooks and O’Malley This content is distributed under the terms of the Creative Commons Attribution 4.0 International license.

### Qualitative analysis of long definitions of dysbiosis.

To gain further insight into what are often sketchy definitions, we selected a portfolio of 100 articles with longer definitions, via several search mechanisms (Table S2). We then analyzed these definitions qualitatively and categorized them. They, too, fitted our three categories but added further dimensions to each. These longer definitions also make “change” and “imbalance” almost equal in terms of how many times such definitions were invoked (48 and 44 instances, respectively). “Change” or alteration is frequently connected to claims about negative consequences for the host. “Imbalance” is very commonly defined as “loss of homeostasis,” but homeostasis itself is rarely elaborated. When it is, it becomes part of a circular definition: imbalance is loss of homeostasis, and homeostasis is a balanced state. The “specific” category (just seven of our long definitions) is elaborated as the bloom of specified pathogens and/or the loss of commensal lineages (not always specified). Definitions of this third sort are then sometimes subordinated to either “change” or “imbalance” categories.

10.1128/mBio.01492-17.3 TABLE S2  List of long definitions. Download TABLE S2, PDF file, 0.1 MB. Copyright © 2017 Hooks and O’Malley. 2017 Hooks and O’Malley This content is distributed under the terms of the Creative Commons Attribution 4.0 International license.

Our qualitative analysis of these 100 longer definitions turned up almost no outlier definitions (just one) compared to the semi-automated analysis of the 554 abstracts. This is probably because we discarded large numbers of texts from our long-definition search due to the papers having no definition at all (dysbiosis being taken for granted, even when being discussed as a major causal factor in an illness) or having only an extremely abbreviated definition (e.g., “alteration in the microbiota” or “imbalance in the microbiota”).

A few articles from our publication sources for 100 long definitions summarize different meanings of dysbiosis (see also Table S3). For example, Levy et al. (2) discover “three types of dysbiosis: bloom of pathobionts, loss of commensals, loss of diversity.” Petersen and Round (20) also discuss three types that may occur together: loss of beneficial microorganisms, the expansion of harmful groups, and a general loss of diversity. Vangay et al. (21) find four types of dysbiosis: “loss of keystone taxa, loss of diversity, shifts in metabolic capacity, and blooms of pathogens.” We consider “bloom of pathobionts” and “loss of keystone taxa” to fit the finer-grained definitions in our “specific” category, whereas loss of commensals and loss of diversity are part of the broad category of “change.” None of these three summaries clearly separates the “imbalance” category, which we found to be quantitatively and qualitatively important. Olesen and Alm (1), whose commentary on dysbiosis was not part of our analysis, also identify this category and consider it a central theme.

10.1128/mBio.01492-17.4 TABLE S3  List of long additional definitions. Download TABLE S3, PDF file, 0.1 MB. Copyright © 2017 Hooks and O’Malley. 2017 Hooks and O’Malley This content is distributed under the terms of the Creative Commons Attribution 4.0 International license.

In addition, Vangay et al. (21) construct a new category of metabolic change. Although most uses of dysbiosis refer to compositional change, a few articles mention functional change as well (e.g., 22). And in particular, an older bridging paper, from the late 1990s, that brings Haenel’s legacy into the microbiome era specifically emphasizes metabolic change as part of dysbiosis (23) (Table S3). Holzapfel et al. argue that “metabolic activity and certain turnover rates may be more important than actual numbers of bacterial species” (23). We will come back to functional accounts of dysbiosis in the section below as we look into the implications of these different definitions.

## IMPLICATIONS OF THESE CATEGORIES

As microbiome research attempts to become more explanatory, a few authors have begun to question the concept of dysbiosis. Olesen and Alm (1) provide a brief but insightful interrogation of their impressions of how the term is used. Our quantitative and qualitative bibliometric analysis allows us to turn a series of criticisms into constructive suggestions for the future of microbiome research.

### (i) Is dysbiosis so broad that it can mean almost any change in microbiota composition?

Broad catchall definitions are not normally esteemed in scientific practice. Too many cases fall under such terms, and they cannot thus discriminate between different and potentially meaningful situations. Due to the known variability between the microbiota of any human and those of others, and limited samples of healthy and ill hosts, dysbiosis is easy to find. The broader the definition, the more easily it is detected. We certainly found dysbiosis identified by this means, and it was not uncommon for any change in microbiota composition to be the main finding of the microbiome analysis as long as it was associated with illness. Some papers attempt to be more discriminatory by limiting dysbiosis to major changes. For example, Antharam et al. (24) classify a microbiota as dysbiotic if it exhibits “a profound alteration of the gut microbiota, or dysbiosis … characterized by markedly decreased biodiversity and species richness” (see Tables S1 and S2 for more examples).

Many of our 100 long definitions specify that for the altered microbiota to be considered dysbiotic, the changed state would need to have a negative effect on the host. Representing this position, Petersen and Round (20) state that “dysbiosis is any change to the composition of resident commensal communities relative to the community found in healthy individuals” (see Table S2 for many similar definitions). Sometimes, this requirement is not stated but is implied either by using pejorative descriptions of change (“aberrant,” “disturbance,” or “unfavorable”) or by immediately connecting “findings” of dysbiosis to a disease outcome. The illness requirement is also specified by suggesting changes that include an increase in pathogens (e.g., references 25 and 26), which are then self-explanatory as agents of illness.

There are some basic problems with such broad illness-linked accounts of dysbiosis. One, as Olesen and Alm (1) argue, is that “the fact that healthy and ill people have different microbiomes is no longer a novel or useful observation,” especially when any sort of difference counts as dysbiosis. Levy et al. (2) suggest that simple comparisons of healthy and diseased people are insufficient and that broader-scale comparisons are necessary (across different forms of the same disease and different diseases altogether). Along with broader cross-sectional sampling, time-series data are indispensable for understanding these relationships (27).

But more generally, there is an overarching methodological problem in these attributions of dysbiosis: by searching for microbiota change in ill people, the dysbiotic state is circularly confirmed as conferring illness. As Bäckhed et al. (28) observe, “current evidence is insufficient to distinguish between dysbiosis as a cause or consequence of the disease.” Yet despite the uncertain role that dysbiosis plays in disease, it is frequently discussed as if its contribution were straightforward, even though mechanisms are unknown.

### (ii) Do these broad and sometimes circular uses of dysbiosis obstruct fine-grained mechanistic analysis?

When microbiome researchers invoke dysbiosis, they can be implying two different scenarios: one in which dysbiosis is reliably coproduced by whatever causes the disease (or by the disease itself), and another in which dysbiosis itself is a major causal factor in producing the disease. If the first holds, dysbiosis is at most diagnostic. If the latter, dysbiosis is explaining the disease. The identification of dysbiosis could, therefore, lead directly to effective treatment. Both diagnostic and explanatory uses of dysbiosis are common, sometimes in the same definition: for example, “an imbalance in the microbiota composition and a shift in their function from normal to disease” (29) and “altered microbiota … sufficient to induce disease” (30) (see Tables S1 and S2 for additional examples)

There is broad consensus in microbiota research that the field needs to move beyond associations to more exact causal factors. Associations may deliver hypotheses about causal relationships, which means that the former are a valuable initial source of insight for explanation and intervention. However, when dysbiosis is invoked as something substantive, it may produce the impression that the mechanism of the disease itself has been discovered. So far, mechanistic discoveries have been few and far between in microbiome research. Assertions of “dysbiosis” tend to function as placeholders, by indicating that microbiome results may be relevant to the illness. Although compositional change may be conceived as a “mechanism,” mechanisms usually need to be more specific and manipulable in order to be testable. It is finding out more precisely the explanatory “place” that dysbiosis is holding that is the ultimate aim of health-related microbiome inquiry.

But if mechanisms of disease are desirable, is taxonomic composition the right focus? Should it be physiological function? Microbiota are not efficiently designed physiological machines. They assemble both stochastically and deterministically. The latter assembly is dictated by environment, including host selection processes. Selection is unlikely to be targeting taxa; it works on traits such as function. Because of this, dysbiosis and nondysbiosis are probably more effectively understood in functional terms (2, 31). The high interindividual variability of microbiomes may be underpinned by stable “core” functions on which different organisms converge due to selection (32). Taxonomic analyses across disease groups are often unable to reveal common patterns of variation, but functional analysis may achieve this, especially if time-series data are available (31, 33).

Although focused mechanistic understandings of host-microbiota interactions will probably depend on both taxonomic and functional analyses of microbiomes, extracting mechanisms from dysbiosis findings is not going to be easy—particularly if culturing is required to understand how pathways bring about particular effects. What may help in streamlining this transition is a better understanding of nondysbiosis. Because any account of dysbiosis relies conceptually on the positive state being disturbed, this has implications for how mechanisms and explanations might emerge from microbiota research.

### (iii) Homeostasis, eubiosis, and normobiosis: what are the implications of the concepts used as opposites of dysbiosis?

Homeostasis is the front-runner term in microbiota research for the desired nondysbiotic state. Classically, homeostasis implies a dynamic process that has mechanisms to return the system to a balanced state after perturbation (e.g., 34). It is exceedingly rare, however, for articles that refer to homeostasis vis-à-vis microbiota to specify the mechanisms by which this supposed rebalancing is achieved, although the immune system is often generally invoked (e.g., 35 and 36). Some researchers suggest that gut microbiota and human hosts have coevolved symbiotically and cooperatively (37), thereby “leading to physiological homeostasis” (35, 38). Little further is said to establish such evolutionary claims, even when it is claimed that evolution will produce an optimal state for hosts and microbiota. On a more ecological basis, Iebba et al. (39) argue for a “Nash equilibrium,” whereby no member of the community is better off when doing anything differently. “Any form of life that is outside [these] rules, or deviates from the equilibrium, inevitably is disadvantaged” (39). Reid et al. (34) suggest that competitive exclusion may be the ecological mechanism for “restoring normal homeostasis” in cases of fecal microbiota transplants to treat *Clostridium difficile* infections.

Following this ecological line of thinking, ideas about stability—a key theoretical concept in ecology—may be relevant when the positive “balanced” or “homeostatic” state of the microbiota is being discussed (32, 40). Diversity is often argued to be the key to stability, balance, and health. Microbiome researchers propose in various ways that when the “delicate balance” of the microbiota is upset by large-scale diversity loss in particular, it can lead to the “chronic disequilibrium referred to as dysbiosis” (38) (see Tables S1 and S2 for more examples). One general explanation is that lower diversity may make hosts in some contexts more vulnerable to the large-scale microbiota alterations that are associated with illness (41).

Greater diversity is not always the key to health, however. As Shade (42) points out, “there are countless examples of ecosystems in which higher diversity is not more meritorious,” including human-microbiota ecosystems (see also 43). Even when it appears as if increasing diversity via fecal microbiota transplants in *C. difficile* patients “cures” them, the success of such transfers probably lies in very narrow functional groups (44). Deeper understanding of the dynamics that produce or undermine stability will probably require modeling of less complex communities (45).

Moya and Ferrer (31) argue not only that dysbiosis should be understood as a “breach of robustness”—meaning a microbiota unable to return to a stable state—but also that stability and instability need functional interpretations that go beyond taxonomic interpretations (31, 46). This is because although microbiota composition may change considerably even over short timescales, functional contributions of the community may remain stable (22, 28). Other researchers, however, note that dysbiosis itself can be understood as a stable state (e.g., 32 and 47). Some elaborations of this view even go so far as to say that stable microbiomes in most healthy people today are probably dysbiotic, because these do not match an earlier “optimally” evolved state of the human microbiota (e.g., 48).

Overall, more time-series data are needed to facilitate ecological explanations of how microbiota stability or “homeostasis” comes about and its relationship to host physiology over time (27, 45, 49–51). Importantly, stability is not necessarily explained by cooperative relationships. In fact, competitive dynamics between all the organisms involved very probably explain stable communities of host and microbiota (52, 53). Any theoretical elaboration of balance or homeostasis in microbiome research would need to take these explanations into account.

Eubiosis, the positive term that Scheunert and Haenel favored, is used in modest but increasing numbers of papers. Usually, eubiosis just means the microbiota in a disease-free host (e.g., 54). A eubiotic state can additionally be characterized as “balanced” between beneficial and harmful bacteria (e.g., 39 and 55). Certain proportions of taxa may consistently be associated with that lack of disease, which then gives reason to believe—as did Haenel—that a quantitative measure of normality can be developed. This is complicated, however, by the growing recognition that composition may not be as important as robust function in maintaining host health.

Normobiosis is a relative newcomer to the terminological scene but is gaining ground on the other two terms. It refers simply to the microbiota patterns found in a healthy host; when the host becomes ill, normobiosis is expected to transform into dysbiosis (e.g., 56 and 57). Disease is thus associated with the loss of normobiosis, and often this loss is measured with reference to particular biomarker taxa (e.g., 58).

All three of these terms (homeostasis, eubiosis, and normobiosis) rely on the idea of a normal healthy microbiome and microbiota. The homeostasis concept implies an explanation of this dynamic normal state, but it is seldom provided; eubiosis and normobiosis are usually intended as static descriptive terms, which is where most unelaborated uses of homeostasis fit. The question that all three terms raise, therefore, is how “normality” is defined and detected.

### (iv) How are distinctions between normal and abnormal microbiota defined?

Earlier intestinal microbiologists believed that quantifying microbiota differences would reveal what causes dysbiosis. They therefore made efforts to calculate “normal” microflora statistically (e.g., 18 and 59). A number of contemporary microbiota researchers have also made attempts to develop dysbiosis indices that can quantify bacterial contributions to the unhealthy state (e.g., 56 and 60; also see Text S1). All these indices may be statistically underpowered, given small sample sizes and human and microbiota variation. A recent high-powered study assessing core microbiota across a large human sample (4,000 European samples and 308 non-European samples) found that associations between many medical conditions and microbiome variation were less robust than expected (61). Occasionally, dysbiotic microbiota are found to have positive effects on host health (e.g., 62).

In general, because microbiota indices are not constructed on the basis of time-series data, they cannot capture responses to perturbation—the very phenomenon that dysbiosis attempts to describe. Moreover, even the best indexing efforts with larger samples (e.g., 60) are tied very tightly to single diseases, whereas what is usually wanted from such an index is more general predictions about health and illness. Given the sorts of comparisons that most studies are making, the capacity to discriminate normal from abnormal microbiota is not going to emerge very soon. In short, dysbiosis, whether quantified or not, remains a promissory note for future work. Just as Haenel worried in the 1980s, it is not clear at all how much work is achieved by claims about dysbiosis (or its opposite).

## CONCLUSIONS AND OUTLOOK

A number of the papers that we analyzed raised concerns about dysbiosis and the conceptual role that it plays in microbiome analyses (e.g., 2 and 22; see also reference 1). Many more papers noted that dysbiosis, although perceived as a real phenomenon, is not clearly established as a cause or an effect (e.g., 2, 28, and [Bibr B63][Bibr B64][Bibr B65]). An emerging theme in a small body of more critical literature questions whether compositional definitions are adequate to the task of establishing microbiota causality in disease. As explained above, a growing number of authors believe that a shift to functional definitions of dysbiosis will be central to more specific causal attributions in microbiota research (e.g., 22, 31, and 33). Whether taxonomic or functional, genuine insights into dysbiosis will require larger samples taken at different time points. All these issues, which come to the fore in our analysis, raise doubts about the current usefulness of dysbiosis to describe and explain research findings. We are not by any means suggesting that dysbiosis be abandoned in microbiome research, but we would urge that its use be reflected on and elaborated to make clear what it is doing.

Continued use of the term dysbiosis can be understood in two ways: as a part of scientific analysis (thus requiring more rigor) or as a broad communication tool (when looseness and broadness may be functional for communicative purposes). Problems arise when these two ways of using language are conflated. Our overview suggests that this may be happening more often than not. As a broad descriptive placeholder (i.e., “something is different here and it may indicate something causal”), dysbiosis works like a flag for future work. But if a loose description like this is viewed as the concluding finding in its own right, then more fine-grained discovery may be preempted.

The historical legacy of dysbiosis is interesting here. Already in the 1960s and 1970s, intestinal microorganism researchers such as Haenel saw the need to quantify claims about dysbiosis and eubiosis in disease (Fig. 2). Despite the increasing frequency with which today’s microbiota researchers refer to dysbiosis, only a few of them follow Haenel’s quantification cue. However, when examining further what the concept of dysbiosis does and could do, it becomes clear that simply quantifying taxa has limitations. By truly engaging with dysbiosis as a conceptual resource, microbiome research may find a way to build on and go beyond its historical predecessors.
