# Supplementary material for: Dysbiosis and Its Discontents
Source: mBio. 2017 Oct 10;8(5):e01492-17. doi: 10.1128/mBio.01492-17 (PMC5635691; doi:10.1128/mBio.01492-17)
Supplement: TEXT S1 [file mbo005173528s1.docx]

**Text S1**

**1. Outline of methodology**

**Quantification of short definitions**

Using Entrez, we downloaded citation records from NCBI Medline for all 10,040 articles that contained the MeSH term “microbiota” (date of access: 30.01.2017). We used MeSH terms as opposed to simple text search to make sure that the papers were all truly in the correct domain of research. An additional benefit is that the MeSH term “microbiota” contains all following key words:

- Microbiotas
- Microbiome
- Microbiomes
- Human Microbiome
- Human Microbiomes
- Microbiomes, Human
- Microbiome, Human

Searching for “microbiota[MeSH]” thus gives exactly the same number of results as “microbiome[MeSH]”.

Within the 10,040 microbiota articles, 9167 had an abstract available. In those abstracts, the word “dysbiosis” was mentioned 867 times, in 843 sentences, in 554 abstracts. We extracted the dates and journal details of these articles from information provided in Medline records.

We manually assigned the definitions of dysbiosis in these 554 abstracts into four categories: imbalance, change, specific and other. We included all explicit definitions, plus implicit definitions when the context of the abstract made clear the intended meaning of dysbiosis. Altogether we ended up with 239 different definitions from our collection of abstracts (Table S1).

**Quantification of long definitions**

To find texts with long dysbiosis definitions we first carried out a Google Scholar search with the terms “dysbiosis and microbiota” and “dysbiosis and microbiome”. We identified the remainder of the texts with long definitions from a PubMed abstract search followed by a manual text check for discussions of dysbiosis in a microbiota research context (Table S2).

Our manual analysis discarded short unqualified definitions (e.g., ‘alteration to the microbiota’, ‘disturbed balance’, ‘loss of homeostasis’), all undefined uses of dysbiosis (even when many details were given about the context in which dysbiosis was found: e.g., ‘dysbiosis promotes inflammation and colitis’), plus the many very specific operationalizations of dysbiosis that implied a definition but did not provide it (e.g., ‘dysbiosis of the Bifidobacterium-Enterococcus ratio’). We also ignored long definitions in unindexed journals and books (found via Google Scholar). Once we had 100 long definitions, we classified them using the same categories we identified for the short definitions. Only one outlier definition was found.

**2. Dysbiosis indices**

Casén et al. (1) define a ‘Dysbiosis Index’ as a score based on the distance from ‘normobiotic reference cohort’, a largely Norwegian sample of 668 people (almost 300 of whom were healthy; the rest had IBD or IBS). This tool calculates the ‘deviation’ of any microbiome from the model normal state.

A similarly large sample forms the basis for Gevers et al.’s (2) ‘Microbial Dysbiosis index’ for Crohn’s disease (CD). This study, based on multi-location samples from 668 patients (including non-IBD controls), formulates the index as the ‘log of [total abundance in organisms increased in CD] over [total abundance of organisms decreased in CD]’ (2, p. 385). Increases and decreases of biomarker groups represent quantitatively the ‘imbalance’ in microbiota that was indicative of disease. The update in 2017 on how the index performs acknowledges it can sort sick and healthy patients (known already, as the division of samples into Crohn’s and non-IBD patients shows) but at least so far ‘cannot inform clinical care’ (3, p. 303).

Other efforts to quantify dysbiosis also examine differences in taxa composition between healthy and ill subjects. Bajaj et al. (4) construct a ‘cirrhosis dysbiosis ratio’ of autochthonous to non-autochthonous taxa that maps microbiome changes during disease progression over almost 250 patients (plus 25 controls). They summarize the index as semi-quantitatively capturing changes in the abundance of ‘good vs. bad bacteria’ (4, p. 914). Jalanka-Tuovinen et al. (5) create their ‘Index of Microbial Dysbiosis’ by analyzing differences between 57 subjects at different stages of irritable bowel syndrome (including 11 healthy controls). This index, based on 27 genus-level groups, worked as a profiling measure for discriminating features of a broad-spectrum disorder and suggesting some possible mechanisms.

Kim et al. (6) worked on a small sample of 18 people with and 18 people without aphthous stomatitis (an oral mucosal disorder) to create a ‘dysbiosis index’ based on the abundance of only two species. They found that a decrease in numbers of a member of the so-called core microbiome and in increase in numbers of a rarer bacterium captured the ‘imbalance’ associated with disease symptoms. Mondot et al. (7) also detected effects from specific groups of bacteria, but additionally found that overall community structure – ‘less organised’ versus ‘tightly correlated and cohesive’ as determined by correlation networks (p. 961) – predicted post-surgical remission rates in 20 subjects after surgery for Crohn’s disease.

**Supplementary references for dysbiosis indices**

1. **Casén C, Vebø HC, Sekelja M, Hegge FT, Karlsson MK, Ciemniejewska E, Dzankovic S, Frøyland C, Nestestog R, Engstrand L, Munkholm P, Nielsen OH, Rogler G, Simrén M, Öhman L, Vatn MH, Rudi K**. Aliment Pharmacol Ther **42**:71–83, 2015.
2. **Gevers D, Kugathasan S, Denson LA, Vázquez-Baeza Y, Van Treuren W, Ren B, Schwager E, Knights D, Song SJ, Yassour M, Morgan XC, Kostic AD, Luo C, González A, McDonald D, Haberman Y, Walters T, Baker S, Rosh J, Stephens M, Heyman M, Markowitz J, Baldassano R, Griffiths A, Sylvester F, Mack D, Kim S, Crandall W, Hyams J, Huttenhower C, Knight R, Xavier RJ**. Cell Host Microbe **15**:382–392, 2014.
3. **Gevers D, Kugathasan S, Knights D, Kostic AD, Knight R, Xavier RJ.** **21**:301–304, 2017.
4. **Bajaj JS, Heuman DM, Hylemon PB, Sanyal AJ, White MB, Monteith P, Noble NA, Unser AB, Daita K, Fisher AR, Sikaroodi M, Gillevet PM**. J Hepatol **60**:940–947, 2014.
5. **Jalanka-Tuovinen J, Salojärvi J, Salonen A, Immonen O, Garsed K, Kelly FM, Zaitoun A, Palva A, Spiller RC, de Vos WM.** Gut **63**:1737–45, 2014.
6. **Kim Y, Choi YS, Baek KJ, Yoon S-H, Park HK, Choi Y**. BMC Microbiol **16 Suppl 1**:57, 2016.
7. **Mondot S, Lepage P, Seksik P, Allez M, Tréton X, Bouhnik Y, Colombel JF, Leclerc M, Pochart P, Doré J, Marteau P, GETAID**. Gut **65**:954–962, 2016.
