# Supplementary material for: Dysbiosis and Its Discontents
Source: mBio. 2017 Oct 10;8(5):e01492-17. doi: 10.1128/mBio.01492-17 (PMC5635691; doi:10.1128/mBio.01492-17)
Supplement: TABLE S1 [file mbo005173528st1.pdf]

Table S1: List of short definitions.

Coding: C = Change, I = Imbalance, S = Specific, O = Other.

Short definitions (quotes)	Reference	type
The HF/HS diet created a specific inflammatory environment in the gut, correlated with intestinal mucosa dysbiosis characterized by an overgrowth of pro-inflammatory Proteobacteria such as E. coli, a decrease in protective bacteria, and a significantly decreased of SCFA concentrations.	Agus et al., Sci Rep 6:19032, 2016.	S
Alterations in microbial composition can result in intestinal dysbiosis, which has been implicated in several diseases including obesity, inflammatory bowel disease, and liver diseases.	Anand et al., Semin Liver Dis 36:37-47, 2016.	C
Dysbiosis, the absence of normal colonization, is associated with many disease conditions.	Bertelsen et al., Best Pract Res Clin Gastroenterol 30:39-48, 2016.	O
Only samples with positive 16S results for L. iners and/or L. crispatus within each group were included in subsequent comparative protein analyses: Lactobacillus crispatus-dominated VMB cluster (with 16S-proven L. iners (ni) = 0, and with 16S-proven L. crispatus (nc) = 5), L. iners-dominated VMB cluster (ni = 11, nc = 4), moderate dysbiosis (ni = 12, nc = 2); and severe dysbiosis (ni = 8, nc = 2).	Borgdorff et al., PLoS One 11:e0150767, 2016.	S
A large number of observational and/or case-control studies of IBD patients have confirmed substantive changes in gut bacterial profiles (dysbiosis) associated with disease.	Burman et al., Dig Dis 34:64-71, 2016.	C
Although treatment protocols, donor selection, stool preparation and delivery methods varied widely, with a few reports following an identical protocol, FMT has diffused to other areas where the alterations of the gut microbiota ecology (or dysbiosis) have been theorized to play a causative role, including inflammatory bowel disease (IBD) and irritable bowel syndrome (IBS), among several other extra-intestinal disorders (i.e. metabolic syndrome and obesity, multiple sclerosis, cardiovascular diseases).	Cammarota et al., Dig Dis 34:279-85, 2016.	C
An astronaut's body during spaceflight encounters increased risk for microbial infections and conditions because of immune dysregulation and altered microbiome, i.e. dysbiosis.	Cervantes et al., Int Rev Immunol 35:67-82, 2016.	C
Furthermore, alterations in the composition or balance of the intestinal microbiota, or dysbiosis, are associated with many gastrointestinal diseases.	Chang et al., Best Pract Res Clin Gastroenterol 30:3-15, 2016.	I

The dysbiosis of gut microbiota indicated by the mean diversity (Shannon index) and mean similarity (Sorenson index) was severe as the liver cirrhosis developed, and AS supplementation had an apparent intervention effect on the dysbiosis of gut microbiota at 4 wk.	Chen et al., World J Gastroenterol 22:2949-59, 2016.	C
CONCLUSIONS: These observations suggest dysbiosis in RA patients resulting from the abundance of certain rare bacterial lineages.	Chen et al., Genome Med 8:43, 2016.	S
The link between the two has become evident, as recent studies have linked intestinal dysbiosis, or the disproportionate balance of beneficial to pathogenic microbes, with increased inflammatory disease susceptibility.	Cook et al., Immunol Cell Biol 94:158-63, 2016.	I
We hypothesize that dysbiosis between regular residents of the upper respiratory tract (URT) microbiome, that is balance between commensals and potential pathogens, is involved in pathogen overgrowth and consequently disease.	de Steenhuijsen Piters et al., ISME J 10:97-108, 2016.	I
While the healthy skin microbiome functions as guardians of host defense, increased or decreased bacterial composition of the skin microbiome (called dysbiosis) leads to skin inflammation and disease.	Di Domizio et al., Rev Med Suisse 12:660-4, 2016.	C
CONCLUSIONS: Our 16S rRNA sequence data demonstrate intestinal dysbiosis at the community level in Korean CD patients, which is similar to alterations of the intestinal microbial community seen in the western counterparts.	Eun et al., BMC Gastroenterol 16:28, 2016.	C
In addition to the known dysfunctions in barrier function of the skin and immunologic disturbances, evidence is rising that frequent skin disorders, e.g. atopic dermatitis, might be connected to a dysbiosis of the microbial community and changes in the skin microbiome.	Fyhrquist et al., Curr Allergy Asthma Rep 16:40, 2016.	C
Indeed, many changes in the bacterial composition of the gut microbiota have been reported in colorectal cancer, suggesting a major role of dysbiosis in colorectal carcinogenesis.	Gagniere et al., World J Gastroenterol 22:501-18, 2016.	C
Our study reveals dysbiosis in the diversity of microbial community structure in lesional skin of vitiligo subjects.	Ganju et al., Sci Rep 6:18761, 2016.	C
These results indicated that dysbiosis of gut microbiota was associated with the pathogenesis of PCOS.	Guo et al., PLoS One 11:e0153196, 2016.	S
High-fat diet (HFD)-induced alteration in the gut microbial composition, known as dysbiosis, is increasingly recognized as a major risk factor for various diseases, including colon cancer.	Higashimura et al., Am J Physiol Gastrointest Liver Physiol 310:G367-75, 2016.	C

In this work, we analyzed these interactions in mice that were mono-associated with six microorganisms that are representative of inflammatory bowel disease (IBD)-associated dysbiosis: the bacteria Bacteroides thetaiotaomicron, adhesive-invasive Escherichia coli (AIEC), Ruminococcus gnavus and Roseburia intestinalis; a yeast used as a probiotic drug, Saccharomyces boulardii CNCM I-745; and another yeast, Candida albicans.	Hoffmann et al., ISME J 10:460-77, 2016.	S
Imbalances in the gut microbiota, known as dysbiosis, can trigger several immune disorders through the activity of T cells that are both near to and distant from the site of their induction.	Honda et al., Nature 535:75-84, 2016.	I
However, disruption of a stable and diverse community, termed "dysbiosis", has been shown to have a profound impact upon health and disease.	Houghton et al., Int J Mol Sci 17:447, 2016.	C
BACKGROUND: Alteration of the gut microbial population (dysbiosis) may increase the risk for allergies and other conditions.	Hua et al., EBioMedicine 3:172-9, 2016.	C
Using a letrozole-induced PCOS mouse model, we demonstrated significant diet-independent changes in the gut microbial community, suggesting that gut microbiome dysbiosis may also occur in PCOS women.	Kelley et al., PLoS One 11:e0146509, 2016.	C
A dysbiosis index, which was developed using the relative abundance of A. johnsonii and S. salivarius and the regression coefficients, correctly predicted 83 % of the total cases for the absence or presence of RAS.	Kim et al., BMC Microbiol 16 Suppl 1:57, 2016.	S
Changes in composition of gut microbiota, called dysbiosis, can trigger systemic inflammation, which is known to be involved in the pathophysiology of HF.	Kitai et al., Curr Heart Fail Rep 13:103-9, 2016.	C
In addition, comparative analysis of the microbial communities revealed that both BPA and a HFD favored the growth of Proteobacteria, a microbial marker of dysbiosis.	Lai et al., Environ Pollut 218:923-930, 2016.	S
Dysbiosis of the intestinal microbiota was also induced, as represented by decreased Firmicutes (relative abundance), increased Proteobacteria and migration of Bacteroidetes from the colon to the jejunum.	Leng et al., Sci Rep 6:22814, 2016.	S
Probiotics are reported to improve microbial balance in the intestinal tract and promote the return to a baseline microbial community following a perturbing event (dysbiosis) such as antibiotic therapy.	Linares et al., Bioengineered 7:11-20, 2016.	I

Here, we make the argument that dysbiosis (life in distress) is ongoing at a micro- and macro-scale and that as a central conduit of health and disease, the immune system and its interface with microbiota is a critical target in overcoming the health challenges of the twenty-first century.	Logan et al., Curr Allergy Asthma Rep 16:13, 2016.	O
Here we used metagenomic analysis to systematically characterize fecal microbiomes of children with and without CF, demonstrating marked CF-associated taxonomic dysbiosis and functional imbalance.	Manor et al., Sci Rep 6:22493, 2016.	I
The numbers of Lactobacillus (L.) iners increase in the case of dysbiosis.	Mendling et al., Adv Exp Med Biol 902:83-93, 2016.	S
Inflammatory bowel diseases are characterised by dysbiosis which is an imbalance between pro- and anti-inflammatory bacteria and a reduction in bacterial diversity.	Schulberg et al., Intern Med J 46:266-73, 2016.	I
BACKGROUND: The global alteration of the gut microbial community (dysbiosis) plays an important role in the pathogenesis of inflammatory bowel diseases (IBDs).	Takahashi et al., Digestion 93:59-65, 2016.	C
Importantly, a disturbed microbiota-host relationship, termed dysbiosis, is now recognized to be the root cause for a growing list of diseases, including colorectal cancer (CRC).	Vipperla et al., Food Funct 7:1731-40, 2016.	O
Chronic alcohol consumption is accompanied by intestinal dysbiosis and bacterial overgrowth, yet little is known about the factors that alter the microbial composition or their contribution to liver disease.	Wang et al., Cell Host Microbe 19:227-39, 2016.	C
Together, these results suggest that long-term exposure to psychological stress induces dysbiosis in the immunodeficient mouse in a strain-specific manner and also that alteration of microbial diversity, which may be related to an altered pattern of immunoglobulin secretion in the gastrointestinal tract, might play a crucial role in the development of chronic stress-induced colitis.	Watanabe et al., PLoS One 11:e0150559, 2016.	C
Careful investigations into the composition and function of these microbial communities have suggested that patients with IBD have an imbalance in their gut microbiota, termed dysbiosis.	Xavier et al., Dig Dis 34:558-65, 2016.	I
The resulting dysbiosis, in turn, stimulates an immune response by activating BdDuox and promoting reactive oxygen species (ROS) production that regulates the composition and structure of the gut bacterial community to normal status by repressing the overgrowth of minor pathobionts.	Yao et al., ISME J 10:1037-50, 2016.	S

In conclusion, glyphosate causes dysbiosis which favors the production of BoNT in the rumen.	Ackermann et al., Curr Microbiol 70:374-82, 2015.	C
Alterations in the composition of the commensal microbiota (dysbiosis) seem to be a pathogenic component of functional gastrointestinal disorders, mainly irritable bowel syndrome (IBS), and might participate in the secretomotor and sensory alterations observed in these patients.	Aguilera et al., Gut Microbes 6:10-23, 2015.	C
Disruption of the ecological equilibrium in the gut (i.e., dysbiosis) has been associated with several pathological processes, including obesity and its related comorbidities, with diet being a strong determinant of gut microbial balance.	Anhe et al., Curr Obes Rep 4:389-400, 2015.	I
The growing evidence that alteration in gut microbiota (dysbiosis) may affect liver pathology may allow for a better understanding of its role in the pathogenesis of NAFLD, help to identify patients at risk of progression, and expose a microbial target for prevention and therapeutic intervention.	Aqel et al., Nutr Clin Pract 30:780-6, 2015.	C
Recent evidence in mice has identified a "critical window" early in life where gut microbial changes (dysbiosis) are most influential in experimental asthma.	Arrieta et al., Sci Transl Med 7:307ra152, 2015.	C
CONCLUSIONS: Dysbiosis, represented by reduction in autochthonous bacteria, is present in both saliva and stool in patients with cirrhosis, compared to controls.	Bajaj et al., Hepatology 62:1260-71, 2015.	C
Gut microbes, however, are sensitive to changes in diet, exposure to antibiotics, or infections, all of which cause transient disruptions in the microbial composition, a phenomenon known as dysbiosis.	Belcheva et al., Bioessays 37:403-12, 2015.	C
In IBD, the development of gut dysbiosis and imbalances in host-microbe relationships contribute to the extent, severity, and chronicity of intestinal inflammation.	Bellaguarda et al., Curr Gastroenterol Rep 17:15, 2015.	I
An "imbalance" of the microbiota, frequently also called a "dysbiosis," has been associated with different diseases in recent years.	Biedermann et al., Eur J Pediatr 174:151-67, 2015.	I
Sometimes this balance is lost and a state of dysbiosis arises, exposing the colon to different metabolic and inflammatory stimuli (according to the microbiota's changing profile).	Borges-Canha et al., Rev Esp Enferm Dig 107:659-71, 2015.	I

The microbiota composition, and the Faecalibacterium prausnitzii / Escherichia coli quantitative relationship as dysbiosis indicator, were studied at baseline [T0], one month [T1], and 3 months [T3] after starting treatment using a polymerase chain reaction-denaturing gradient gel electrophoresis [PCR-DGGE] of 16S rRNA gene fragments and quantitative PCR, respectively, in rectal mucosal biopsies from 15 CD patients and four healthy subjects.	Busquets et al., J Crohns Colitis 9:899-906, 2015.	S
Overall, 165 healthy controls (normobiotic reference collection) were used to develop a dysbiosis model with a bacterial profile and Dysbiosis Index score output.	Casen et al., Aliment Pharmacol Ther 42:71-83, 2015.	C
Indican urinary concentration is one of the most common and easily assessable markers of intestinal dysbiosis.	Cassani et al., Parkinsonism Relat Disord 21:389-93, 2015.	C
Our data indicate that WEGL not only reverses HFD-induced gut dysbiosis-as indicated by the decreased Firmicutes-to-Bacteroidetes ratios and endotoxin-bearing Proteobacteria levels-but also maintains intestinal barrier integrity and reduces metabolic endotoxemia.	Chang et al., Nat Commun 6:7489, 2015.	S
Here, we show that dysbiosis of the intestinal microbiota, characterized by an expansion of the Gammaproteobacteria, is tightly linked to age-onset intestinal barrier dysfunction in Drosophila.	Clark et al., Cell Rep 12:1656-67, 2015.	S
Here we report, for the first time, that a peculiar dysbiosis of the gut microbiota is present in patients with Behcet syndrome and this corresponds to specific changes in microbiome profile.	Consolandi et al., Autoimmun Rev 14:269-76, 2015.	C
Studies have shown that excessive alcohol consumption impacts the intestinal microbiota composition, causing disruption of homeostasis (dysbiosis).	Couch et al., PLoS One 10:e0119362, 2015.	I
Quantitative and qualitative alterations in the composition of the gut microbiome could lead to pathological dysbiosis, and have been related to an increasing number of intestinal and extra-intestinal diseases.	D'Argenio et al., Clin Chim Acta 451:97-102, 2015.	C
The analysis of selective fecal bacterial substrates as a measure of alterations in the gut microbiota may be a potential marker of dysbiosis.	De Leoz et al., J Proteome Res 14:491-502, 2015.	C
Analysis of this 'dysbiosis' enables the detection of alterations in specific bacteria, clusters of bacteria or bacterial functions associated with the occurrence or evolution of type 2 diabetes; these bacteria are predominantly involved in the control of inflammation and energy homeostasis.	Delzenne et al., Diabetologia 58:2206-17, 2015.	C

CONCLUSIONS: Patients who have chronic HIV infection and are receiving suppressive ART display intestinal dysbiosis associated with increased microbial translocation and significant associations between specific taxa and markers of microbial translocation and systemic inflammation.	Dinh et al., J Infect Dis 211:19-27, 2015.	O
Dysbiosis or dysbacteriosis is defined as a shift in the intestinal microbiota composition resulting in an imbalance between beneficial and harmful bacteria.	Ducatelle et al., Animal 9:43-8, 2015.	I
Despite advances in our knowledge of changes in microbial composition associated with different health conditions the functional aspects of the oral microbiome that lead to dysbiosis remain for the most part unknown.	Duran-Pinedo et al., Microbes Infect 17:505-16, 2015.	C
Such stimulation is subject to dysregulation during inflammation and disease, contributing to 'dysbiosis' or an abnormal microbiota composition that has been associated with a variety of immune-mediated inflammatory disorders, including celiac disease.	Elson et al., Dig Dis 33:131-6, 2015.	C
Herein, we evaluated whether fecal levels of alpha-defensins could be surrogate marker of intestinal dysbiosis.	Eriguchi et al., Transpl Infect Dis 17:702-6, 2015.	C
The alteration of the homeostasis between physiologic and pathogenic bacteria of intestinal flora causes a condition called dysbiosis.	Frosali et al., J Immunol Res 2015:489821, 2015.	I
It is now accepted that CD is due to a breakdown of immune tolerance (dysbiosis) to bacteria in the intestine.	Fry et al., Exp Dermatol 24:241-4, 2015.	O
However, changes in the composition of the gut microbiota (dysbiosis) may be associated with several clinical conditions, including obesity and metabolic diseases, autoimmune diseases and allergy, acute and chronic intestinal inflammation, irritable bowel syndrome (IBS), allergic gastroenteritis (e.g., eosinophilic gastroenteritis and allergic IBS), and necrotizing enterocolitis.	Goulet et al., Nutr Rev 73 Suppl 1:32-40, 2015.	C
Bifidobacterium/Enterococcus ratio, proposed as a measure of pre-LT gut dysbiosis, was significantly related to the MELD score following the adjustment for the absolute Bifidobacterium (sbeta = -0.333; P = 0.029) and Enterococcus (sbeta = -0.966; P = 0.003) numbers.	Grat et al., Transpl Infect Dis 17:174-84, 2015.	S
We recently reported that alpha-mangostin (alpha-MG), the most abundant xanthone in mangosteen fruit, altered the intestinal microbiome, promoted dysbiosis, and exacerbated colitis in C57BL/6J mice.	Gutierrez-Orozco et al., Nutrients 7:764-84, 2015.	C

Therefore, many diseases and conditions could be impacted by the gut microbiota when its composition is imbalanced, otherwise known as dysbiosis.	Ho et al., BMC Immunol 16:21, 2015.	I
Here, we show that Aim2(-/-) mice are highly susceptible to dextran sodium sulfate-induced colitis that is associated with microbial dysbiosis as represented by higher colonic burden of commensal Escherichia coli.	Hu et al., Cell Rep 13:1922-36, 2015.	S
Regulation of sialic acid catabolism opens new perspectives for the treatment of intestinal inflammation as manifested by E. coli dysbiosis.	Huang et al., Nat Commun 6:8141, 2015.	S
Dysbiosis refers to a state of imbalance among the colonies of microorganisms within the body, which brings abnormal increase of specific minor components and decrease in the normally dominant species.	Iizasa et al., World J Gastroenterol 21:11450-7, 2015.	I
The Firmicutes to Bacteroidetes ratio was significantly decreased in both UC and CD compared with controls, indicative of a dysbiosis in both conditions.	Kabeerdoss et al., Indian J Med Res 142:23-32, 2015.	S
Alterations of this ecology (or dysbiosis) have been implicated in a number of disease states, and the prototypical example is Clostridium difficile infection (CDI).	Kelly et al., Gastroenterology 149:223-37, 2015.	C
If PD patients have altered colonic microbiota, dysbiosis might be the mechanism of neuroinflammation that leads to alpha-Syn misfolding and PD pathology.	Keshavarzian et al., Mov Disord 30:1351-60, 2015.	C
Alterations in the homeostasis of the microbiota are considered dysbiosis.	Kim et al., J Clin Gastroenterol 49 Suppl 1:S20-4, 2015.	I
The human diseases, obesity and inflammatory bowel disease, are prime examples of dysbiosis.	Kim et al., J Clin Gastroenterol 49 Suppl 1:S20-4, 2015.	O
Most commensal bacteria (i.e. Psychrobacter, Prevotella, and Faecalibacterium) in the healthy gastrointestinal tract were decreased due to dysbiosis induced by PEDV infection.	Koh et al., Microbes Environ 30:284-7, 2015.	S
This dysbiosis leads to an increase in inflammatory reactions in which staphylococcal toxins play an important role.	Lacour et al., Ann Dermatol Venereol 142 Suppl 1:S18-22, 2015.	S
When this microbial ecosystem becomes disrupted, the health of the human host can suffer; a condition called dysbiosis.	Larsen et al., Gigascience 4:42, 2015.	O
Intestinal dysbiosis, with altered levels of specific bacteria, is consistently seen in CRC.	Leung et al., Expert Rev Gastroenterol Hepatol 9:651-7, 2015.	C
Abnormal composition of intestinal bacteria–"dysbiosis"-is characteristic of Crohn's disease.	Lewis et al., Cell Host Microbe 18:489-500, 2015.	C
The Firmicutes/Bacteroidetes ratio was increased significantly, which indicated that dysbiosis of the oral microbiota participated in the process of HBV-CLD development.	Ling et al., Sci Rep 5:17098, 2015.	S

Changes in these factors can cause microbiome disruption known as dysbiosis, leading to the outgrowth of potential pathogenic bacteria or a decrease in the number of beneficial bacteria.	Mankowska-Wierzbicka et al., Postepy Hig Med Dosw (Online) 69:978-85, 2015.	C
While some results related to dysbiosis in IBD are different between studies owing to variations of sample type, method of investigation, patient profiles, and medication, the most consistent observation in IBD is reduced bacterial diversity, a decrease of Firmicutes, and an increase of Proteobacteria.	Matsuoka et al., Semin Immunopathol 37:47-55, 2015.	C
In addition, microbiota from mucosal samples represented the underlying dysbiosis, whereas fecal samples seem not to be appropriate to detect shifts in microbial composition.	Mira-Pascual et al., J Gastroenterol 50:167-79, 2015.	S
Various uremic toxins are derived from gut microbiota, and an imbalance of gut microbiota or dysbiosis is related to renal failure.	Mishima et al., J Am Soc Nephrol 26:1787-94, 2015.	I
Correcting the dysbiosis and altered gut microbiota might deserve consideration as a potential strategy for the prevention and treatment of MS.	Miyake et al., PLoS One 10:e0137429, 2015.	C
Because numerous reports have indicated the importance of gut microbiota in the regulation of weight gain, it is reasonable to speculate that AN patients might have a microbial imbalance, i.e. dysbiosis, in their gut.	Morita et al., PLoS One 10:e0145274, 2015.	I
This may be on account of distinct changes in the gut microbiota termed as dysbiosis.	Mukhopadhya et al., Microbes Infect 17:304-10, 2015.	C
SUMMARY: A better understanding of the mechanisms linking HFD to alterations in gut microbiota is necessary to allow for the regulation of dysbiosis and ensuing promotion of antiobesity effects.	Murphy et al., Curr Opin Clin Nutr Metab Care 18:515-20, 2015.	C
In addition, although the colon microbiota did not show a clear bacterial dysbiosis among the three conditions, the abundance of some particular bacteria was changed with respect to normal controls.	Murugesan et al., Eur J Clin Microbiol Infect Dis 34:1337-46, 2015.	C
OBJECTIVE: HIV-1 infection is characterized by altered intestinal barrier, gut microbiota dysbiosis, and systemic inflammation.	Nowak et al., AIDS 29:2409-18, 2015.	C
A predominance of A. vaginae, G. vaginalis and L. iners with a concomitant paucity of L. crispatus in the cervical microbiota was associated with CIN risk, suggesting that bacterial dysbiosis and its combination with oncogenic HPV may be a risk factor for cervical neoplasia.	Oh et al., Clin Microbiol Infect 21:674.e1-9, 2015.	S

OBJECTIVE: Intestinal dysbiosis has been associated with coeliac disease (CD), but whether the alterations are cause or consequence of the disease is unknown.	Olivares et al., Gut 64:406-17, 2015.	C
Disruption of these relationships and the structure of the bacterial communities that inhabit the gut can contribute to dysbiosis, leading to disease.	Pacheco et al., Microbiol Spectr 3:, 2015.	O
RECENT FINDINGS: Multiple studies have demonstrated airway microbiota dysbiosis, characterized by Proteobacteria expansion in the lower airways, to be a consistent trait of established adult asthma.	Panzer et al., Curr Opin Rheumatol 27:373-80, 2015.	S
Liver cirrhosis is a paradigm of intestinal dysbiosis.	Ponziani et al., World J Gastroenterol 21:12322-33, 2015.	O
The chief origin of these microbes is the gut microbiome (especially when it shifts composition to a pathogenic state, known as 'dysbiosis').	Potgieter et al., FEMS Microbiol Rev 39:567-91, 2015.	C
Dysbiosis' is also used to describe translocation of cells into blood or other tissues.	Potgieter et al., FEMS Microbiol Rev 39:567-91, 2015.	O
This microbial dysbiosis in dominant phyla was significantly prevented in chitosan administrated HSD group.	Prajapati et al., Curr Pharm Biotechnol 17:173-84, 2015.	C
In children with functional dyspepsia the analysis established dysbiosis alterations in intestinal biotope, characterizing by deficiency of bifidobacteria and selection of opportunistic microorganisms.	Rakova et al., Klin Lab Diagn 60:50-3, 2015.	S
These findings indicate that dysbiosis observed in murine models of colitis is associated with changes in the composition of bacteria present in the oral cavity and in saliva.	Rautava et al., J Gastroenterol Hepatol 30:521-7, 2015.	C
Although it is clear that an imbalance or dysbiosis in the microbiota is associated with disease, its interrelatedness to disease penetrance is largely unknown.	Ray et al., Immunology 146:359-68, 2015.	I
The etiology and the pathogenesis of IBS are still not clear; however, recent studies have implicated a role for alterations in the intestinal microbiota (dysbiosis) in the pathophysiology of the disorder.	Ringel et al., J Clin Gastroenterol 49 Suppl 1:S56-9, 2015.	C
Thus, these sorghum brans may protect against alterations observed during colitis including reduced microbial diversity and richness, and dysbiosis of Firmicutes/Bacteroidetes.	Ritchie et al., FEMS Microbiol Ecol 91:fiv008, 2015.	S
CONCLUSIONS: The abundance of some taxa in the faecal microbiota of diarrhoeic horses can be a result of microbiome dysbiosis, and therefore a cause of intestinal disease, or some of these taxa may act as equine enteric pathogens.	Rodriguez et al., BMC Microbiol 15:181, 2015.	C

There is also a growing recognition that disruption of commensal microbiota, a phenomenon known as dysbiosis, is associated with several common disorders, including inflammatory bowel disease, type 2 diabetes and oncogenesis.	Rogers et al., Intern Med J 45:889-98, 2015.	C
There are mounting reports in animal models and human epidemiologic studies linking disruptive alterations in the gut microbiota or dysbiosis and ASD symptomology.	Rosenfeld et al., Drug Metab Dispos 43:1557-71, 2015.	C
We postulate that the 'confrontation' between the 'evil' alliance and 'benign' alliance and the shifting balance between them may be responsible for dysbiosis of the milk microbiome that permits mastitis.	Sam et al., Sci Rep 5:8275, 2015.	I
Furthermore, CD patients have imbalances in the intestinal microbiota (dysbiosis), which are not fully normalized despite their adherence to a gluten-free diet.	Sanz et al., Ann Nutr Metab 67 Suppl 2:28-41, 2015.	I
We also propose that an increased prevalence of Proteobacteria is a potential diagnostic signature of dysbiosis and risk of disease.	Shin et al., Trends Biotechnol 33:496-503, 2015.	S
In CD or UC patients, an abnormally composed microbiota, referred to as "dysbiosis," is commonly observed (discussed later).	Stecher et al., Microbiol Spectr 3: doi:10.1128/microbiolspec.MBP-0008-2014, 2015.	C
This dysbiosis was accompanied by changes in bacterial functional gene categories.	Suchodolski et al., PLoS One 10:e0127378, 2015.	C
Notably, an apparent dysbiosis occurred, characterized by a reduction of Firmicutes and an increase in Proteobacteria.	Taniguchi et al., Sci Rep 5:15699, 2015.	S
This has adverse effects on the commensal gut microbial community, as it disrupts the intricate balance between specific bacterial groups within this ecosystem, potentially leading to dysbiosis.	Tulstrup et al., PLoS One 10:e0144854, 2015.	I
In premature infants, alterations in the intestinal microbiota (dysbiosis) are associated with risk of necrotizing enterocolitis (NEC) and sepsis, and the influence of HMOs on the microbiota is unclear.	Underwood et al., Pediatr Res 78:670-7, 2015.	C
Perturbations of the microbial composition (dysbiosis) and reduced diversity may promote disease susceptibility and recurrence.	van Best et al., Birth Defects Res C Embryo Today 105:240-51, 2015.	C
Antibiotic use during infancy induces imbalances in gut microbiota, called dysbiosis.	Vangay et al., Cell Host Microbe 17:553-64, 2015.	I
We recommend future studies into the microbiome-mediated effects of antibiotics focused on four types of dysbiosis: loss of keystone taxa, loss of diversity, shifts in metabolic capacity, and blooms of pathogens.	Vangay et al., Cell Host Microbe 17:553-64, 2015.	O
Dysbiosis, or alteration of this microbiome, can result in Clostridium difficile infection and may play a role in other conditions.	Vindigni et al., Expert Rev Clin Immunol 11:781-3, 2015.	C

When microbiota homeostasis is impaired and dysbiosis occurs, the malfunction of epithelial barrier leads to intestinal and systemic disorders, chiefly immunologic and metabolic.	Virili et al., Endocrine 49:583-7, 2015.	I
Perturbation of gut microbial composition, termed dysbiosis, is associated with an increased susceptibility to intestinal pathogens and is a hallmark of a number of inflammatory, metabolic, and infectious diseases.	Vong et al., Am J Physiol Gastrointest Liver Physiol 309:G181-92, 2015.	C
Among a genetically susceptible host, the shift of gut microbiota (or 'dysbiosis') can lead to increasing the susceptibility to IBD.	Wang et al., Curr Opin Gastroenterol 31:277-82, 2015.	C
Some of the changes that were noted have been associated with 'dysbiosis' and proinflammatory states in other species, so it is possible that subclinical alteration in the intestinal microbiota could influence the health of FIV-infected cats.	Weese et al., Vet Microbiol 180:96-102, 2015.	C
CONCLUSION: FMT can be a preferential measure to restore the dysbiosis caused by MSRA enterocolitis.	Wei et al., BMC Infect Dis 15:265, 2015.	S
BACKGROUND: The human intestinal microbiota is a key regulator of host metabolic and immune functions and alterations in the microbiome ('dysbiosis') have been implicated in several human diseases.	Wieland et al., Aliment Pharmacol Ther 42:1051-63, 2015.	C
This dysbiosis was correlated with the severity of the disease.	Yin et al., J Am Heart Assoc 4:e002699, 2015.	S
Altered microbial composition (dysbiosis) has been correlated with a number of diseases in humans.	Alegre et al., Am J Transplant 14:1236-48, 2014.	C
Altered intestinal microbiota (dysbiosis) may stimulate hepatic fat deposition through several mechanisms: regulation of gut permeability, increasing low-grade inflammation, modulation of dietary choline metabolism, regulation of bile acid metabolism and producing endogenous ethanol.	Arslan et al., World J Gastroenterol 20:16452-63, 2014.	C
Moreover, these emerging characteristics would be consistent with the polymicrobial synergy and dysbiosis (PSD) periodontal pathogenesis model.	Aruni et al., J Dent Res 93:725-32, 2014.	S
Studies documenting an altered microbiome associated with EAC and its precedents suggest that dysbiosis may be contributing to carcinogenesis, potentially mediated by interactions with toll-like receptors.	Baghdadi et al., Clin Lab Med 34:721-32, 2014.	C
The ratio of autochthonous to non-autochthonous taxa was calculated as the cirrhosis dysbiosis ratio (CDR); a low number indicating dysbiosis.	Bajaj et al., J Hepatol 60:940-7, 2014.	C

In this phase I study, Lactobacillus GG is safe and well-tolerated in cirrhosis and is associated with a reduction in endotoxemia and dysbiosis.	Bajaj et al., Aliment Pharmacol Ther 39:1113-25, 2014.	S
This suggests that microbiota changes observed in dysbiosis, obesity, or antibiotic therapy may affect the cross talk between hematopoiesis and the microbiota, potentially exacerbating inflammatory or infectious states in the host.	Balmer et al., J Immunol 193:5273-83, 2014.	C
The dysbiosis theory of inflammatory bowel disease (IBD) posits that there is an alteration in the gut microbiome as an important underpinning of disease etiology.	Bernstein et al., Nestle Nutr Inst Workshop Ser 79:83-100, 2014.	C
More importantly, regarding previously described microbial hallmarks of dysbiosis in inflammatory bowel diseases, a variety of observed microbial alterations after smoking cessation deserve further consideration in view of the divergent effect of smoking on the clinical course of Crohn's disease and ulcerative colitis.	Biedermann et al., Inflamm Bowel Dis 20:1496-501, 2014.	C
Host diet is known to alter microbiota composition, implying that dietary treatments might alleviate diseases arising from altered microbial composition ('dysbiosis').	Bolnick et al., Nat Commun 5:4500, 2014.	C
Dysbiosis, i.e. imbalance of the intestinal microbiome, may have a role in the progression of NAFLD.	Buffet et al., Bull Acad Natl Med 198:1641-52, 2014.	I
Antibiotics, prebiotics and probiotics are the best known and commercially available options to overcome gastrointestinal dysbiosis.	Cammarota et al., Intern Emerg Med 9:365-73, 2014.	C
Supplementation of n-3PUFAs can partially counteract such gut dysbiosis, lower endotoxin level in portal vein blood, and improve the body weight.	Cao et al., Zhongguo Yi Xue Ke Xue Yuan Xue Bao 36:496-500, 2014.	S
Moreover, numerous independent studies reported a dysbiosis, i.e., a modification of intestinal microbiota composition, with an imbalance between the abundance of beneficial and harmful bacteria.	Carriere et al., World J Gastroenterol 20:12102-17, 2014.	I
An imbalance of the normal enteric microbiota composition (termed dysbiosis) underlies the pathogenesis of UC.	Chen et al., World J Gastroenterol 20:15657-63, 2014.	I
An altered balance between microbiota and its host (dysbiosis) would appear to contribute to the development of Inflammatory Bowel Disease (IBD), Crohn's Disease (CD) and Ulcerative Colitis (UC).	Comito et al., Ital J Pediatr 40:32, 2014.	I
Both diseases appear to result from a perturbation among relatively minor constituents in local microbial communities resulting in dysbiosis.	Costalonga et al., Immunol Lett 162:22-38, 2014.	C
Microbiota composition (dysbiosis) was evaluated by Pyrosequencing.	De Minicis et al., Hepatology 59:1738-49, 2014.	O

Cyclooxygenase 2 expression started to increase 4 d after DSS withdrawal ($P < 0.05$), when dysbiosis had recovered, and continued to increase during the recovery phase.	De Fazio et al., World J Gastroenterol 20:2051-61, 2014.	S
Whether they contribute to dysbiosis, i.e., the departure from microbiota composition in symbiosis at equilibrium and entry into a state favoring human or animal disease is unknown at present.	De Paepe et al., Front Cell Infect Microbiol 4:39, 2014.	I
Fecal microbial transplantations appear to be promising therapies for dysbiosis-associated diseases; however, probiotic microorganisms have been growing in popularity due to increasing numbers of studies proving that certain strains present health promoting properties, among them the beneficial balance of the intestinal microbiota.	de Moreno de LeBlanc et al., World J Gastroenterol 20:16518-28, 2014.	I
Metagenomic analysis showed an important dysbiosis in CF gut microbiota associated with a high concentration of Proteobacteria.	del Campo et al., J Cyst Fibros 13:716-22, 2014.	S
We propose that exacerbations are occasions of respiratory tract dysbiosis–a disorder of the respiratory tract microbial ecosystem with negative effects on host biology.	Dickson et al., Lancet 384:691-702, 2014.	C
Shifts in the bacterial composition of the human gut microbiota (i.e. dysbiosis) have been associated with digestive tract dysfunctions such as inflammatory bowel diseases.	Duca et al., Front Horm Res 42:73-82, 2014.	C
However, there is mounting evidence that dysbiosis, a state of pathological imbalance in the gut microbiome is present in many disease states.	Dulal et al., Cancer J 20:225-31, 2014.	I
The overrepresented microbes have previously been associated with the potential to cause disease under certain conditions, illustrating that conventionalisation proceeds through a transient state that resembles situations associated with dysbiosis.	El Aidy et al., Benef Microbes 5:67-77, 2014.	S
Furthermore, perturbation of the microbiota-host symbiosis (dysbiosis) is considered a common pathogenic mechanism connecting gastrointestinal, ocular and neuropsychiatric symptoms.	Feher et al., Orv Hetil 155:1454-60, 2014.	C
Changes to this structure can be injurious to the health of the host, a concept termed dysbiosis.	Galley et al., Gut Microbes 5:748-60, 2014.	C
Intestinal dysbiosis, as modeled using GF mice (containing no microbiota), bacterial infection with an enteric pathogen, and administration of probiotics, can modulate cognitive behavior including learning and memory.	Gareau et al., Adv Exp Med Biol 817:357-71, 2014.	C

Moreover, there is mounting evidence that our microbiomes change us, by promoting health through their beneficial actions or by increasing our susceptibility to diseases through a process termed dysbiosis.	Gerber et al., FEBS Lett 588:4131-9, 2014.	C
The selective flourishing of inflammophilic bacteria can perpetuate inflammatory tissue destruction by setting off a 'vicious cycle' for disease progression, in which dysbiosis and inflammation reinforce each other.	Hajishengallis et al., Mol Oral Microbiol 29:248-57, 2014.	C
Strikingly, siblings shared aspects of intestinal dysbiosis with patients with CD (lower concentrations of Faecalibacterium prausnitzii (p=0.048), Clostridia cluster IV (p=0.003) and Roseburia spp.	Hedin et al., Gut 63:1578-86, 2014.	S
This dysbiosis is reflected, based on in silico functional inference, in an overrepresentation of oxidative phosphorylation and glycan utilization pathways in SLE patient microbiota.	Hevia et al., MBio 5:e01548-14, 2014.	S
This altered microbiome or "dysbiosis" is a key player in the protracted course of inflammation in IBD.	Hold et al., World J Gastroenterol 20:1192-210, 2014.	C
Vaccine responsiveness may be improved by promoting intestinal bifidobacteria and minimizing dysbiosis early in infancy.	Huda et al., Pediatrics 134:e362-72, 2014.	S
Genetic alteration in immune/epithelial function can affect host gardening of the intestinal microbiome, contributing to the diversity of intestinal microbiota within a population and in some cases allowing for unfavorable microbial ecologies (dysbiosis) that confer disease susceptibility.	Jacobs et al., FEBS Lett 588:4102-11, 2014.	O
RESULTS: Microbiota analysis revealed a bacterial profile of 27 genus-like groups, providing an Index of Microbial Dysbiosis (IMD), which significantly separated patient groups and controls.	Jalanka-Tuovinen et al., Gut 63:1737-45, 2014.	S
Dysbiosis, defined as a pathological imbalance in a microbial community, is becoming increasingly appreciated as a 'central environmental factor' that is both associated with complex phenotypes and affected by host genetics, diet and antibiotic use.	Jones et al., Expert Opin Biol Ther 14:467-82, 2014.	I
Dramatic changes in the environment, resulting in the dysregulated composition of intestinal microbiota or dysbiosis, may be associated with the fundamental causes of IBD.	Kanai et al., Korean J Intern Med 29:409-15, 2014.	C
Increasing evidence has pointed to intestinal dysbiosis as a potential factor in a genetically susceptible individual.	Kao et al., J Clin Gastroenterol 48:625-8, 2014.	C

Changes in these factors can cause microbiome disruption known as dysbiosis, leading to the outgrowth of potential pathogenic bacteria or decrease in the number of beneficial bacteria.	Karczewski et al., Autoimmunity 47:494-504, 2014.	C
Furthermore, connections are now being made between microbiota dysbiosis and a variety of different diseases such as rheumatoid arthritis, inflammatory bowel disease, type 1 diabetes, atopy, and obesity.	Keeney et al., Annu Rev Microbiol 68:217-35, 2014.	O
The signs of III degree dysbiosis, by reducing the concentration of Bacteroides spp.	Kuchmak et al., Lik Sprava (12):63-5, 2014.	S
Alterations in the microbiota, also termed dysbiosis, seem to be involved in the pathogenesis of a variety of intestinal and extraintestinal diseases.	Kump et al., Z Gastroenterol 52:1485-92, 2014.	C
Intestinal dysbiosis refers to a state where living metazoans harbor harmful intestinal microflora.	Lee et al., Dev Comp Immunol 42:102-10, 2014.	O
CONCLUSION: Our findings demonstrate that mucosal lymphocyte depletion leads to the dysbiosis of gut fungal microbiota, suggesting its role in maintaining host-fungus homeostasis.	Li et al., Transplantation 98:951-9, 2014.	S
The Firmicutes/Bacteroidetes ratio was increased significantly in patients with CDAD, which indicated that dysbiosis of faecal microbiota was closely associated with CDAD.	Ling et al., Sci Rep 4:7485, 2014.	S
However, the composition and diversity of the gut microbiome can be readily affected by external factors, which raises the possibility that exposure to toxic environmental chemicals leads to gut microbiome alteration, or dysbiosis.	Lu et al., Environ Health Perspect 122:284-91, 2014.	C
Decompensated dysbiosis was registered in 71.9% of patients in this group which manifested in increased quantitative indicators of transient microflora crop with pathogenic characteristics and lack of microflora with protective characteristics.	Lytvynenko et al., Lik Sprava (5-6):82-7, 2014.	C
Additionally, conventionalization of germ-free mice with intestinal contents from alcohol-fed conventional mice induced injury and inflammation in both the liver and the intestine, suggesting that alcohol intake successively caused a perturbation of the intestinal microbiota (dysbiosis) and liver injury.	Canesso et al., BMC Microbiol 14:240, 2014.	C
RESULTS: HF/HS diet led to dysbiosis in WT and transgenic CEABAC10 mice, with a particular increase in E coli population in HF/HS-fed CEABAC10 mice.	Martinez-Medina et al., Gut 63:116-24, 2014.	S

A small and poorly defined role for dysbiosis in the development of IBS symptoms has been established through characterization of altered intestinal microbiota in IBS patients and reported improvement of subjective symptoms after its manipulation with prebiotics, probiotics, or antibiotics.	Mayer et al., Gastroenterology 146:1500-12, 2014.	C
Mediterranean Diet also favours the prevalence of saccharolytic species, while Western Diet promotes the shift towards a proteolytic profile (dysbiosis).	Montemurno et al., Kidney Blood Press Res 39:114-23, 2014.	C
In addition, the expression of certain genes correlated with GMC dysbiosis, i.e., low FPrau-to-Bacteroides ratio.	Munukka et al., J Hepatol 61:132-8, 2014.	S
Dysbiosis, changes in microbiome structure, has been linked to inflammatory, functional and metabolic disorders such as IBD, IBS and obesity.	Panda et al., Endocr Metab Immune Disord Drug Targets 14:290-9, 2014.	C
Although the intestinal microbial community exists in a state of homeostasis called eubiosis, environmental and genetics factors can lead to microbial perturbation or dysbiosis, a state associated with various pathologies including inflammatory bowel diseases (IBD) and colorectal cancer (CRC).	Perez-Chanona et al., Bioessays 36:658-64, 2014.	I
Thus, perturbations to the structure of complex commensal communities (referred to as dysbiosis) can lead to deficient education of the host immune system and subsequent development of immune mediated diseases.	Petersen et al., Cell Microbiol 16:1024-33, 2014.	C
Infection of the gastrointestinal tract is commonly linked to pathological imbalances of the resident microbiota, termed dysbiosis.	Pham et al., Curr Opin Microbiol 17:67-74, 2014.	I
Many diseases are associated with changes in the microbiota, called dysbiosis.	Pifer et al., Microbiol Spectr 2: doi:10.1128/microbiolspec.EHEC-0015-2013, 2014.	C
Resveratrol improves the gut microbiota dysbiosis induced by the HF diet, including increasing the Bacteroidetes-to-Firmicutes ratios, significantly inhibiting the growth of Enterococcus faecalis, and increasing the growth of Lactobacillus and Bifidobacterium.	Qiao et al., Food Funct 5:1241-9, 2014.	S
CONCLUSIONS: In this work, we report that CD-associated dysbiosis, characterized by a decrease in Firmicutes, correlates with the time-to-relapse after infliximab withdrawal.	Rajca et al., Inflamm Bowel Dis 20:978-86, 2014.	S
When this control is lost, dysbiosis, i.e. deregulation in bacterial communities, can occur and this can lead to inflammatory disorders, including inflammatory bowel disease, obesity, diabetes and autism.	Rescigno et al., Cell Microbiol 16:1004-13, 2014.	O

This dysbiosis was characterized by an expansion of segmented filamentous bacteria, associated with altered intestinal production of IL-22 and IgA, and was transmissible to wild-type mice, resulting in increased susceptibility to DSS.	Roberts et al., J Immunol 193:5249-63, 2014.	S
The dominant genera, Streptococcus, Prevotella, Neisseria, Haemophilus, Veillonella, and Gemella, were found to largely contribute to dysbiosis (dysbacteriosis) observed in the salivary microbiota of IBD patients.	Said et al., DNA Res 21:15-25, 2014.	S
Abnormal profiles of fecal and mucosally associated enteric bacteria (dysbiosis) occur in Crohn's disease, UC, pouchitis and experimental enterocolitis, with a proliferation of aggressive species that promote experimental colitis and a corresponding decrease in protective bacterial subsets.	Sartor et al., Nestle Nutr Inst Workshop Ser 79:29-39, 2014.	C
Gut microbial imbalance (dysbiosis), has been linked to important human diseases such as inflammation related disorders.	Schippa et al., Nutrients 6:5786-805, 2014.	I
Dysbiosis results in intestinal inflammation, a breakdown of the intestinal barrier, and translocation of microbial products in animal models.	Schnabl et al., Gastroenterology 146:1513-24, 2014.	I
Dysbiosis, the alteration of the complex ecologic system of gut microbes, is associated with and causally responsible for multiple types of pathologies.	Serino et al., Curr Cardiol Rep 16:540, 2014.	C
Alterations in composition and function of the microbiota, termed dysbiosis, have been implicated in a multitude of metabolic and inflammatory diseases in humans.	Shapiro et al., Curr Opin Immunol 30:54-62, 2014.	C
However, this commensal community exists in careful balance that, if disrupted, enters dysbiosis; this has been shown to contribute to the pathogenesis of colon, gastric, esophageal, pancreatic, laryngeal, breast, and gallbladder carcinomas.	Sheflin et al., Curr Oncol Rep 16:406, 2014.	I
The imbalance in the composition of the microbiota (dysbiosis) observed in IBD patients is one of the strongest arguments and provides the rationale for a therapeutic manipulation of the gut microbiota.	Sokol et al., Dig Dis 32 Suppl 1:10-7, 2014.	I
Differentiating between an optimal microbiota, one that increases disease risk, and one that is causative or potentiates disease will be required to further understand both the etiology and possible treatments for health problems related to microbiota dysbiosis.	Sonnenburg et al., Cell Metab 20:779-86, 2014.	O

We identify NAS-altered microbial metabolic pathways that are linked to host susceptibility to metabolic disease, and demonstrate similar NAS-induced dysbiosis and glucose intolerance in healthy human subjects.	Suez et al., Nature 514:181-6, 2014.	C
Alterations of the intestinal microbiota can occur by changes in composition (dysbiosis), function, or microbiota-host interactions and they can be directly correlated with several diseases.	Tojo et al., World J Gastroenterol 20:15163-76, 2014.	C
The development of IBD is often associated with qualitative and quantitative disorders of the intestinal microbial flora (dysbiosis).	Tomasello et al., World J Gastroenterol 20:18121-30, 2014.	C
These studies have now conclusively shown that lactobacilli-dominated VMB are associated with a healthy vaginal micro-environment and that bacterial vaginosis (BV) is best described as a polybacterial dysbiosis.	van de Wijgert et al., PLoS One 9:e105998, 2014.	C
Many studies have reported a dysbiosis in IBD, characterised by a decrease in diversity, a decreased abundance of some dominant commensal members (such as Clostridium IV and XIVa) and an increase in detrimental bacteria (such as sulphate reducing bacteria and Escherichia coli).	Verbeke et al., Proc Nutr Soc 73:490-7, 2014.	C
Furthermore, a largely neglected area of research activity has been the role of live probiotic cultures that contribute to repairing dysbiosis (a leaky gut barrier abnormality) in the gastrointestinal tract (GIT).	Vitetta et al., Inflammopharmacology 22:333-9, 2014.	O
The results indicated intestinal dysbiosis in patients with persistent symptoms even while adhering to a strict GFD.	Wacklin et al., Am J Gastroenterol 109:1933-41, 2014.	S
However, a microbial imbalance (dysbiosis) characterized by a decreased abundance of Clostridia and a bloom of facultative anaerobic Proteobacteria is commonly observed during inflammation in the large bowel.	Winter et al., Cell Microbiol 16:179-84, 2014.	I
Transformation of the microbiome in precursor states to esophageal adenocarcinoma-reflux esophagitis and Barrett metaplasia-from a predominance of gram-positive bacteria to mostly gram-negative bacteria raises the possibility that dysbiosis is contributing to pathogenesis.	Yang et al., Cancer J 20:207-10, 2014.	C
Microbiome analysis has identified a state of microbial imbalance (dysbiosis) in patients with chronic intestinal inflammation and colorectal cancer.	Yang et al., Dis Model Mech 7:1131-42, 2014.	I
Biological properties that confer stability in the microbiome are important for the prevention of dysbiosis-a microbial shift toward a disease, e.g., periodontitis or caries.	Zaura et al., Front Cell Infect Microbiol 4:85, 2014.	C

This study investigated the influence of a probiotic treatment on the survival and microbiota of brook charr (Salvelinus fontinalis), focusing on its disturbance of the natural microbiota (dysbiosis).	Boutin et al., Can J Microbiol 59:662-70, 2013.	S
Various disease states are associated with an imbalance of protective and pathogenic bacteria in the gut, termed dysbiosis.	Chan et al., Ann Nutr Metab 63 Suppl 2:28-40, 2013.	I
Several stressors like infestations of Varroa mites and the use of pesticides can contribute to the occurrence of dysbiosis phenomena, resulting in a perturbation of the microbiocenosis established in the honeybee body.	Crotti et al., N Biotechnol 30:716-22, 2013.	I
The microbial dysbiosis of the IBS gut microbiota (more sulfate-reducing bacteria and Enterobacteriaceae and less bifidobacteria) could be maintained in gnotobiotic rats.	Crouzet et al., Neurogastroenterol Motil 25:e272-82, 2013.	S
Deciphering these microbial signatures and their metabolites that govern short and long-term equilibrium, as well as imbalances in host-microbial relationships, may provide novel diagnostic tools and/or therapeutic targets for specific disorders associated with intestinal dysbiosis and loss of homeostasis.	El Aidy et al., Bioessays 35:913-23, 2013.	I
Thereby, these approaches can provide novel diagnostic tools and therapeutic targets, or either of the two, in humans for specific disorders associated with intestinal dysbiosis and loss of homeostasis.	El Aidy et al., Curr Opin Gastroenterol 29:621-7, 2013.	I
This dysbiosis in inflammasome-deficient mice has a profound impact on their physiology and pathophysiology, both locally in the intestine and systemically.	Elinav et al., Methods Mol Biol 1040:185-94, 2013.	C
Dysbiosis has been associated with a series of gastrointestinal disorders that include non-alcoholic fatty liver disease, celiac disease, and irritable bowel syndrome.	Icaza-Chavez et al., Rev Gastroenterol Mex 78:240-8, 2013.	C
Low-grade inflammation of the intestine results in metabolic dysfunction, in which dysbiosis of the gut microbiota is intimately involved.	Kim et al., Environ Microbiol Rep 5:765-75, 2013.	I
We discuss the concept of dysbiosis, proposing that the functional composition of the gut microbiome may provide a more consistent definition of dysbiosis and may more readily provide evidence of genome-microbiome interactions in future exploratory studies.	Knights et al., Gut 62:1505-10, 2013.	C
BACKGROUND: In patients with ulcerative colitis (UC), alterations of the intestinal microbiota, termed dysbiosis, have been postulated to contribute to intestinal inflammation.	Kump et al., Inflamm Bowel Dis 19:2155-65, 2013.	C

In addition, we show that dysbiosis of the lingual microbiome in SIV infection is characterized by outgrowth of Gemella morbillorum that may result from impaired macrophage function.	Ocon et al., PLoS One 8:e80863, 2013.	S
Multiomic analysis of microbiome dysbiosis has identified important CDI-associated microbial community shifts that may form the basis of future targeted bacteriotherapy, and functional metabolite biomarkers that require further characterization.	Peniche et al., Curr Opin Infect Dis 26:447-53, 2013.	C
CONCLUSIONS: Dysbiosis of mucosal microbiota occurs in CD with decreases of richness and biodiversity.	Ran et al., Zhonghua Yi Xue Za Zhi 93:2884-9, 2013.	C
When the composition of this microbiota is unfavorably altered, termed dysbiosis, the host is rendered more susceptible to a variety of chronic diseases.	Salzman et al., Semin Immunol 25:334-41, 2013.	C
Alerted bacterial profile, known as dysbiosis precedes development of allergy in children.	Strzepa et al., Postepy Hig Med Dosw (Online) 67:908-20, 2013.	C
However, the high complexity and low inter-individual overlap of intestinal microbial composition are formidable barriers to identifying microbial taxa representing this dysbiosis.	Tong et al., PLoS One 8:e80702, 2013.	C
Whether the contribution to colon carcinogenesis is generated through the presence of an overall dysbiosis or by specific pathogens is still a matter for debate.	Turner et al., Curr Gastroenterol Rep 15:346, 2013.	C
The dysbiosis of fecal microbiota, characterized by the enrichment of potential pathogens and the decrease in butyrate-producing members, may therefore represent a specific microbial signature of CRC.	Wu et al., Microb Ecol 66:462-70, 2013.	S
Aberrant gut microbial composition, termed 'dysbiosis', has been reported in inflammatory bowel disease patients who are at increased risk for CRC development.	Yang et al., Trends Mol Med 19:714-25, 2013.	C
This suggests strategies for treating dysbiosis of the microbiota and associated inflammatory disorders.	Yu et al., Glycobiology 23:1281-92, 2013.	S
The function of microbial colonization in establishing immune system homeostasis has been reported, whereas host-microbe interactions and genetically determined variation of stratum corneum properties might be linked to skin dysbiosis.	Zeeuwen et al., Curr Opin Allergy Clin Immunol 13:514-20, 2013.	I
T2DM-related dysbiosis was observed, including the separation of microbial communities and a change of alpha diversity between the different glucose intolerance statuses.	Zhang et al., PLoS One 8:e71108, 2013.	C

Thus, P. gingivalis creates a dysbiosis between the host and dental plaque, and this may represent one mechanism by which periodontitis can be initiated.	Darveau et al., J Dent Res 91:816-20, 2012.	S
One of the core requirements for a potentially pathogenic community to arise involves the capacity of certain species, termed 'keystone pathogens', to modulate the host response in ways that impair immune surveillance and tip the balance from homeostasis to dysbiosis.	Hajishengallis et al., Mol Oral Microbiol 27:409-19, 2012.	I
Relapsing C. difficile disease in humans is linked to a pathological imbalance within the intestinal microbiota, termed dysbiosis, which remains poorly understood.	Lawley et al., PLoS Pathog 8:e1002995, 2012.	I
We show that mice infected with epidemic C. difficile (genotype 027/BI) develop highly contagious, chronic intestinal disease and persistent dysbiosis characterized by a distinct, simplified microbiota containing opportunistic pathogens and altered metabolite production.	Lawley et al., PLoS Pathog 8:e1002995, 2012.	O
