# Supplementary material for: Dysbiosis and Its Discontents
Source: mBio. 2017 Oct 10;8(5):e01492-17. doi: 10.1128/mBio.01492-17 (PMC5635691; doi:10.1128/mBio.01492-17)
Supplement: TABLE S2 [file mbo005173528st2.pdf]

Table S2: List of long definitions.

Coding: C = Change, I = Imbalance, S = Specific, O = Other.

Long definitions (quotes)	Reference	type
'an alteration of the gut microbiota... which is characterized by a reduced abundance of obligate anaerobic bacteria and an expansion of facultative Proteobacteria such as commensal E. coli' (p. 208)	Hughes et al., Cell Host Microbe 21:208-219, 2017.	S
'a compositional and functional alteration in the microbiota that is driven by a set of environmental and host-related factors that perturb the microbial ecosystem to an extent that exceeds its resistance and resilience capabilities' (pp. 1-2) 'Dysbiosis typically features one or more of the following non-mutually exclusive characteristics: Bloom of pathobionts... Loss of commensals... Loss of diversity' (pp. 2-3)	Levy et al., Nat Rev Immunol 17:219-232, 2017.	C
Quantitation of 'microbial imbalance to disease... calculated as the log of [total abundance in organisms increased in CD] over [total abundance of organisms decreased in CD]' (p. 303)	Gevers et al., Cell Host Microbe 21:301-304, 2017.	S
'compositional or functional shift within host-associated microbial communities that has the potential to facilitate growth of pathogens and/or onset diseases' (p. 888)	Arnold et al., Trends Microbiol 24:887-901, 2016.	C
'an altered microbiota... that manifests as a change in taxonomic composition and/or microbial a-diversity, which are often correlated with disease outcomes' (p. 575)	Arrieta et al., Cell Host Microbe 19:575-578, 2016.	C
'when gut microbiota are disrupted, the resulting immunological dysregulation may lead to a variety of disorders... these observations explain why microbiota dysbiosis may be at the source of a broad range of diseases... abnormalities associated with an altered microbiome' (p. 25)	Buret,, Am J Gastroenterol 3:24-31, 2016.	C
'alterations in the composition or balance of the intestinal microbiota... associated with many gastrointestinal diseases' (p. 3)	Chang et al., Best Pract Res Clin Gastroenterol 30:3-15, 2016.	C
'resulting from the abundance of certain rare bacterial lineages' (p. 1)	Chen et al., Genome Med 8(1):43, 2016.	C
'broad patterns... such as a loss of microbial diversity and increasing representation of gram-negative facultative anaerobes, such as Enterobacteriaceae' (pp. 173-174)	Khanna et al., J Infect Dis 214:173-181, 2016.	S
'reduced diversity or changes in composition, often with an increase in the ratio of pathogenic to commensal organisms' (p. 1024)	Liddicoat et al., BioScience 66:1023-1034, 2016.	C

'perturbations to the structure of complex commensal microbial communities [that] can threaten symbiosis and involve the loss of beneficial microorganisms, the expansion of potentially harmful microbes and/or the loss of overall microbial diversity' (p. 4)	Logan et al., Curr Allergy Asthma Rep 16:13, 2016.	C
loss of normal, health promoting" bacteria allowing overgrowth of disease-promoting pathogenic bacteria... which in turn makes patients susceptible to [illness]' (p. 1)"	McDonald et al., mSphere 1(4):e00199-16, 2016.	C
'A disturbance or imbalance in a biological system, for example, changes in the types and numbers of bacteria in the gut which may lead to developing different diseases' (p. 331)	Marchesi et al., Gut 65:330-339, 2016.	I
'an imbalanced intestinal microbial community with quantitative and qualitative alterations in the composition and metabolic activities of the gut microbiota' (p. 484)	Ramezani et al., Am J Kidney Dis 67:483-498, 2016.	I
'altered pathogenic bacteria in the gut... a state in which intestinal flora have qualitative and quantitative changes in their metabolic activity and local distribution, when compared with a 'normal' functioning gut' (p. 472)	Wing et al., Exp Physiol 101.4:471-477, 2016.	C
'an imbalance in the microbiome structure that results from an abnormal ratio of commensal and pathogenic bacterial species' (p. 3)	Belizario and Napolitano, Front Microbiol 6:1050, 2015.	I
'deviations from normobiosis can result in a transient or permanent microbiotic imbalance known as dysbiosis, which has been linked to several disorders' (p. 72)	Casen et al., Aliment Pharmacol Ther 42:71-83, 2015.	I
'Dysbiosis or dysbacteriosis is defined as a shift in the intestinal microbiota composition resulting in an imbalance between beneficial and harmful bacteria... more specifically a decrease in the relative numbers of beneficial microbes and bloom of harmful microbes in the intestinal tract' (p. 43)	Ducatelle et al., Animal 9:1:43-48, 2015.	I
'a breakdown of immune tolerance (dysbiosis) to microbiota of the intestine' (p. 242)	Fry et al., Exp Dermatol 24:241-244, 2015.	I
'microbiomes lacking commensal species necessary for appropriate physiological and immune development and subsequent maintenance of immune homeostasis' (p. 596)	Fujimura and Lynch, Cell Host Microbe 5:592-602, 2015.	I
'a perturbation within the fragile ecosystem of the microbiome' (p. 3)	Gritz and Bhandari, Front Pediatr 3:17, 2015.	C
'When this microbial ecosystem becomes disrupted... [with] a negative effect on the health of the human host' (p. 1)	Larsen and Dai, Gigascience 4:1-16, 2015.	C
'Aberrant intestinal microbiota composition and function... suggested to associate with many diseases' (p. 1428)	Levy et al., Cell 163:1428-1443, 2015.	C

altered" gut bacterial composition... commonly characterized by an expansion of Proteobacteria and a decrease in Firmicutes along with a decrease in community richness' (p. 489)"	Lewis et al., Cell Host Microbe 18:489-500, 2015.	S
'when pathological imbalances in gut bacterial colonies precipitate disease' (p. 2)	Sagar et al., Gastroenterol Res Pract 2015:398585, 2015.	I
'an imbalanced gut microbiota often arises from a sustained increase in abundance of the phylum Proteobacteria [which is] abnormal [and leads to] a compromised ability to maintain a balanced gut community... an imbalance in the taxonomic composition of gut microbiota [is] called dysbiosis' (p. 496)	Shin et al., Trends Biotechnol 33:496-503, 2015.	I
'a microbiota community associated with a diseased state that can be differentiated from the microbiota community associated with a healthy control state' (Table 1)	Schreiner et al., Curr Opin Gastroenterol 31:69-75, 2015.	C
'four types of dysbiosis: loss of keystone taxa, loss of diversity, shifts in metabolic capacity, and blooms of pathogens' (p. 553)	Vangay et al., Cell Host Microbe 17:553-564, 2015.	C
'the concept that compositional alterations away from the conventional symbiotic gut microbiota may be associated with pathology within the host' (p. 1052)	Wieland et al., Aliment Pharmacol Ther 42:1051-1063, 2015.	C
'a change in relative composition of the different microbe scompared to normal, during a disease state' (p. 65)	Williams and Gallo, Curr Allergy Asthma Rep 15:65, 2015.	C
'alterations in the composition of the human gut microbiome... often characterized by a high abundance of bacteria in the phylum Firmicutes, and... accompanied by an increase of bacteria belonging to the phylum Proteobacteria and fewer members of the Bacteroidetes. Low bacterial diversity is another hallmark of dysbiosis' (p. 111)	Youmans et al., Gut Microbes 6:110-119, 2015.	S
'significant shifts in the microbiota and dominance of bacteria, with enhanced invasive and inflammatory properties that can directly exacerbate inflammation and tissue damage' (p. 130) 'the outgrowth of opportunistic clades of bacteria... the loss of benign fermenting bacteria that produce keystone" metabolites' (p. 131)"	Belkaid and Hand, Cell 157:121-141n, 2014.	C

'dysbiosis results from perturbations of the intestinal microbiota from dietary or environmental changes, certain antibiotic therapies, psychosocial stress, exposure to pathogenic organisms or by altered/dysregulated immune responses... In the majority of perturbations, changes of microbiota composition (both in diversity and abundance) are transient... Gut dysbiosis can, however, also be considered a stable state because certain dominant species may change the gut environment or out-compete other species... The presence of a single pathogenic species (e.g., Escherichia coli), however, may not signify dysbiosis' (p. 163)	Blumstein et al., Evol Med Public Health 2014:163, 2014.	C
'a condition in which the microflora becomes unbalanced and the symbiotic relationship between the host and microbiome is lost' (p. 237)	Chen and Schnabl, Gut Liver 8:237-241, 2014.	I
'disorder and dysregulation of the microbial ecosystem... coupled with a dysregulated host immune response that results in negative effects on host biology' (p. 697)	Dickson et al., Lancet 384:691-702, 2014.	C
'an imbalance of microbial population dynamics... characterized by decreased beneficial commensals/symbionts, overexpression of pathogenic microbiota such as genotoxic bacteria, invasive and inflammation triggering microbiota, procarcinogenic bacteria and cancer enhancing bacterial antigens and metabolites' (Figure 1)	Dulal and Keku, Cancer J 20:225-231, 2014.	I
'unbalanced microbial community... associated with a dysregulated immune response' (p. 382)'an overall drop in species richness and an alteration in the abundance of several taxa' (p. 383	Gevers et al., Cell Host Microbe 15:382-392, 2014.	I
imbalance in the relative abundance or influence of microbial species within the ecosystem (as compared to health), leading to alterations in the host-microbial crosstalk sufficient to mediate destructive inflammation' (p. 249)	Hajishengallis, Mol Oral Microbiol 29:248-257, 2014.	I
'a state in which former commensals behave as proinflammatory pathobionts'	Hajishengallis, Trends Immunol 35:3-11, 2014.	C
'an imbalance of the different microbial entities in the intestine with a disruption of symbiosis... [that] can present as quantitative (intestinal bacterial overgrowth) and qualitative changes in the intestinal microbiota' (p. 763)	Hartmann et al., Alcohol Clin Exp Res 39:763-775, 2015.	I
'disturbance that shifts the composition of the normal microbial community [such that] there is an increase in facultative anaerobic bacteria, which can include potentially harmful microorganisms that induce inflammatory processes... associated with a number of diseases, including inflammatory disorders' (p. 311)	Irrazabal et al., Mol Cell 54:309-320, 2014.	C

'unfavorable microbial ecologies (dysbiosis) that confer disease susceptibility' (p. 4102)	Jacobs et al., FEBS Lett 588:4102-4111, 2014.	C
'a pathological imbalance in a microbial community' (p. 468)	Jones et al., Expert Opin Biol Ther 14:467-482, 2014.	I
'a pathological imbalance in a microbial community... hereditary dysbiosis... loss of maternal transmission of microbes to their offspring (i.e., vertical transmission) as well as [loss of] transmission of microbes through fecally contaminated drinking and bathing water (i.e., horizontal transmission)' (p. 452)	Jones et al., Gut Microbes 5:446-457, 2014.	I
'perturbations affecting the complexity and stability of the microbiome... Features of an imbalanced microbiome include, for example, an increase in gram-negative bacteria linked to an environment of oxidative stress and inflammation and metabolite production' (p. 1490)	Kostic et al., Gastroenterology 146:1489-1499, 2014.	I
'a state where living metazoans harbor harmful intestinal microflora' (p. 102)	Lee and Lee, Dev Comp Immunol 42:102-110, 2014.	O
'an imbalance between protective and harmful bacteria' (p. 1276)	Machiels et al., Gut 63:1275-1283, 2014.	I
'a state characterized by imbalances in the relative abundance or influence of microbial species within an ecosystem' (p. 768)	Maekawa et al., Cell Host Microbe 15:768-778, 2014.	I
'an alteration or disruption of the normal microbiota (bacterial or fungal species) due to exposure of a disruptive factor (such as antibiotics, chronic disease, stress, medical procedures or medications, etc)... there is no current standard definition of 'normal' microbiota' (p. 3)	McFarland, BMJ Open 4:e005047, 2014.	C
'a pathological imbalance in a microbial ecological niche, leading to a skewed overall metabolic signature, as well as low system resilience' (p. 1147)	Mao and Franke, Proteomics 15:1142-1151, 2014.	I
'the loss of a significant set of genera associated with controls in the HIV cases, typical of a dysbiotic microbiome. In many instances these are genera that are thought to be beneficial such as Bacteroides' (p. 11)	Mutlu et al., PLoS Pathog 10(2):e1003829, 2014.	S
'a change in the normal microbiota, or of misrecognition of the normal microbiota within different body environments' (p. 2)	Nibali et al., J Oral Microbiol 6:22962, 2014.	C

'any change to the composition of resident commensal communities relative to the community found in healthy individuals' (p. 1024) 'categorize it into three types... (i) loss of beneficial microbial organisms, (ii) expansion of pathobionts or previously harmful microorganisms, (iii) loss of overall microbial diversity... These three types of dysbiosis are not mutually exclusively [sic] and may all occur concurrently' (p. 1025)	Petersen and Round, Cell Microbiol 16:1024-1033, 2014.	C
'a reduction in bacterial biodiversity that results in fewer bacteria with anti-inflammatory properties and/or more bacteria with proinflammatory properties compared with healthy controls' (p. 978)	Rajca et al., Inflamm Bowel Dis 20:978-986, 2014.	C
'microbiota of... patients significantly differ[s] from that of healthy controls' (p. 16)	Said et al., DNA Res 21:15-25, 2014.	C
'this commensal community exists in careful balance that, if disrupted, enters dysbiosis and contributes to host disease processes, including cancer' (p. 1)	Sheflin et al., Curr Oncol Rep 16(10):406, 2014.	I
'unintentional or maladaptive changes... implicated... in a growing list of Western diseases' (p. 779)	Sonnenburg and Sonnenburg, Cell Metab 20:779-786, 2014.	C
'qualitative and quantitative disorders of the intestinal microbial flora [also known as] dismicrobism' (p. 18122)	Tomasello et al., World J Gastroenterol 20:18121-18130, 2014.	C
'the microbial community is thrown off balance, and the host is prone to systemic infection with enterococci' (p. 426)	Van Tyne and Gilmour, Cell Host Microbe 16:425-427, 2014.	I
'a profound alteration of the gut microbiota... characterized by markedly decreased biodiversity and species richness' (p. 2889)	Antharam et al., J Clin Microbiol 51:2884-2892, 2013.	C
'a decrease in blood bacterial DNA content and an increase in the proportion of Proteobacteria phylum within blood microbiota' (p. 3)	Amar et al., PLoS One 8(1):e54461, 2013.	S
'alterations to the normal homeostasis of gut microbiota... shift from healthy state of being' (p. 1)	Bested et al., Gut Pathog 5:4, 2013.	C
disturbance in normal microbiota of the gut can lead to an imbalance of host-microbe relationships... [this] imbalance exerts adverse effects on the host' (p. 53)	Bien et al., Therap Adv Gastroenterol 6:53-68, 2013.	I
'Imbalances in the composition of the intestinal microbiota' (p. 797)	Buffie and Pamer, Nat Rev Immunol 13:790-801, 2013.	I
'Instability in the composition of gut bacterial communities... disturbances of this balanced state [of coexistence]' (p. 700)	Couturier-Maillard et al., J Clin Invest 123:700-71.1, 2013.	I
'more sulfate-reducing bacteria and Enterobacteriaceae and less bifidobacteria... compared with healthy subjects' (p. e272)	Crouzet et al., Neurogastroenterol Motil 25(4):e272-282, 2013.	S

'disruption of the normal balanced state of the gut... involves the abnormal accumulation or increased virulence of certain commensal populations of bacteria, thereby transforming former symbionts into pathobionts"' (p. 329)"	Kamada et al., Nat Rev Immunol 13:321-335, 2013.	C
'an imbalance in the microbial composition and a shift in their function from normal to disease' (p. 75)	Kim et al., Pediatr Gastroenterol Hepatol Nutr 16:71-79, 2013.	I
'a severe imbalance in the composition of the gut microbiome is often referred to as dysbiosis, but the term is poorly defined... we may continue to find dysbiosis challenging to define in terms of taxonomic or phylogenomic composition... Given its relative stability, the functional composition of the microbiome may provide a more consistent definition of dysbiosis' (p. 1057)	Knights et al., Gut 62:1505-1510, 2013.	I
'Alteration of the microbiota in comparison to the normal, healthy state. Dysbiosis refers to a condition of microbial imbalances compared with the 'eubiosis' condition, a state of equilibrium between bacterial symbionts and pathobionts. Dysbiosis is mostly reported in the digestive tract or on the skin, but can also occur on any exposed surface or mucous membrane. Dysbiosis can be observed at several analytical levels, from diversity to compositional or functional imbalances' (p. 155)	Lepage et al., Gut 62:146-158, 2013.	I
'perturbations of distinct, extremely complex and delicately balanced host-microbe ecosystems' (p. 1)	Ocon et al., PLoS One 8(11):e80863, 2013.	I
'A state of microbial composition that is characterized by an unbalanced proportion of bacteria compared with the proportion in a healthy state' (p. 804)	Schwabe and Jobin, Nat Rev Cancer 13:800-812, 2013.	I
'the disturbed microbiome ecology secondary to external pressures such as host diseases, medications, diet and genetic conditions often leading to abnormalities of the host immune system' (p. 1)	Shapira et al., ISRN Oncol 2013:693920, 2013.	C
'an imbalance in the structural and/or functional configuration of the microbiota leading to a disruption of host-microorganism homeostasis' (p. 235)"	Sommer and Backhed, Nat Rev Microbiol 11:227-238, 2013.	I
'an imbalanced microbial community (including bacteria, yeast, viruses and parasites) characterized by quantitative and qualitative changes in the composition of the microbiota itself, in its modified metabolic activities, or in the local distribution of its members. During a state of dysbiosis, the microbiota is prone to invasions and to blooms of pathogens' (p. 278)	Stecher et al., Nat Rev Microbiol 11:277-284, 2013.	I

'A shift in the balance of microbiota composition such that it may become deleterious to host health' (p. 75) 'situations where microbial composition and activities are shifted from their normal, benign/beneficial, state to another that is more deleterious to host health' (p. 76)	Walker and Lawley, Pharmacol Res 69:75-86, 2013.	C
'characterized by the enrichment of potential pathogens and the decrease in butyrate-producing members' (p. 462)	Wu et al., Microb Ecol 66:462-470, 2013.	C
'an alteration of the microbial community structure associated with disease' (p. 118)	Wu et al., Anaerobe 24:117-120, 2013.	C
'a significant shift in the composition of the gut microbiome in patients... compared to that in healthy controls' (p. 1) 'highlighted by major shifts in bacterial populations from a wide range of taxonomic groups' (p. 3)	Zackular et al., mBio 4(6):e00692-13, 2013.	C
'an imbalance between protective vs. injurious bacteria' (p. 315)	Albenberg et al., Curr Opin Gastroenterol 28(4):314-320, 2012.	I
'The concept of dysbiosis arose from [disease] studies, and altered microbial communities were defined as dysbiotic in the setting of the disease. However, current evidence is insufficient to distinguish between dysbiosis as a cause or consequence of the disease' (p. 612)	Backhed et al., Cell Host Microbe 12:611-622, 2012.	C
'change profoundly the composition of the microbiota in a manner that may be detrimental to the host' (p. 1)	Blumberg and Powrie, Sci Transl Med 4:137rv7, 2012.	C
'A compositional change in the microbiota and/or an abnormality in the interactions between the host and the commensal microbiota' (p. 740)	Collins et al., Nat Rev Microbiol 10:735-742, 2012.	C
'immune dysregulation, loss of microbiome diversity, and colonization with enteric pathogens' (p. 469) 'abnormal Firmicutes composition' (p. 471)	Duplessis et al., Infection 40:469-472, 2012.	C
'unbalancing of the relative abundance of individual components of the microbiota compared with their abundancies in health' (Fig. 1 legend)	Hajishengallis et al., Nat Rev Microbiol 10:717-725, 2012.	I
'an imbalance between enteric microbial colonies and its associated deleterious effects on the colonized host' (p. 4)	Hartmann et al., Front Physiol 3:402, 2012.	I
'altered microbiota... sufficient to induce disease... could thus be considered "dysbiotic"... mice [with] an altered microbiota with increased abundance of members of the Bacteroidetes phylum... dysbiosis alone is sufficient to drive the intestinal inflammation' (p. 1270)	Hooper et al., Science 336:1268-1273, 2012.	C

'a distinct, simplified microbiota containing opportunistic pathogens and altered metabolite production... pathological imbalance within the intestinal microbiota' (p. 1)	Lawley et al., PLoS Pathog 8(10):e1002995, 2012.	I
'shifts in the composition of the microbiota, whether induced by dietary changes, antibiotic treatment or invasive pathogens, can disturb the balance of organisms in the microbiota and alter the metabolic network of the collective to favour the outgrowth of potentially pathogenic constituents. Referred to as dysbiosis, such changes in the microbiota can perturb immune regulatory networks that normally restrain intestinal inflammation, and may contribute to immune-mediated disease directed against antigens of the microbiota' (p. 231)	Maynard et al., Nature 489:231-241, 2012.	I
'imbalance in the intestinal bacteria leading to disease' (p. 1262)	Nicholson et al., Science 336:1262-1267, 2012.	I
'a decrease in the abundance of some universal butyrate-producing bacteria and an increase in various opportunistic pathogens, as well as an enrichment of other microbial functions conferring sulphate reduction and oxidative stress resistance' (p. 55)	Qin et al., Nature 490:55-60, 2012.	C
'the outgrowth, or loss, of certain components of microbiota correlates with intestinal disease... pathogenic changes have been found upon breakdown of host immune homeostasis mechanisms... and in humans there can be similar dysbiosis following treatment with antibiotics... it has been possible to identify individual commensal components associated with or responsible for these effects, such as the loss [or gain of specific bacteria] (p. 107)	Ivanov and Littman, Curr Opin Microbiol 14:106-114, 2011.	I
'alterations in the relative proportions of the phyla constituting the microbial community... linked to specific diseases' (p. 526)	Hamdi et al., J Appl Entomol 135:524-533, 2011.	C
'skewing of the commensal community... triggered not only by pathogenic microbes, but also by otherwise harmless commensal microbes or those that are normally held in check by the microbial ecosystem and/or the metabolic state and immune response of the host... disturbance of this homeostasis' (p. 311)	Littman and Pamer, Cell Host Microbe 10:311-323, 2011.	I
'An imbalance in the bacterial population at a naturally occurring site of colonization, often resulting in health problems' (p. 29)	Reid et al., Nat Rev Microbiol 9:27-38, 2011.	I
'a composition... in which one or a few potentially harmful microorganisms are dominant, thus creating a disease-prone situation' (p. 377)	Santos et al., Trends Food Sci Technol 22:377-385, 2011.	C

'A shift in the relative abundances of the microbial taxa compared with the abundances that are observed in healthy animals... associated with an expanding list of chronic diseases' (p. 279)	Spor et al., Nat Rev Microbiol 9:279-290, 2011.	C
'multi-species assemblages in an overall imbalance between harmful and protective bacteria' (p. 2)	Walker et al., BMC Microbiol 11:7,, 2011.	I
'an overgrowth of pathobionts or a loss of beneficial commensal bacteria' (p. 271)	Chow and Mazmanian, Cell Host Microbe 7:265-276, 2010.	C
'Disruption of the delicate balance between the host and its intestinal microbiota' (p. 850) 'Breakdown of this mutualistic relationship, or dysbiosis, results in perturbation of host function' (p. 851)	Collins et al., Dig Liver Dis 41:850-853, 2009.	I
'alterations in the development or composition of the microbiota [that] disturb the partnership between the microbiota and the human immune system, ultimately leading to altered immune responses that may underlie various human inflammatory disorders' (p. 314)	Round and Mazmanian, Nat Rev Immunol 9:313-323, 2009.	C
'gut microbiota imbalance associated with host diseases' (p. 1183)	Sokol et al., Inflamm Bowel Dis 5:1183-1189., 2009.	I
