# Supplementary material for: Dysbiosis and Its Discontents
Source: mBio. 2017 Oct 10;8(5):e01492-17. doi: 10.1128/mBio.01492-17 (PMC5635691; doi:10.1128/mBio.01492-17)
Supplement: TABLE S3 [file mbo005173528st3.pdf]

Table S3: List of long additional definitions.

Additional definitions, mentioned by many of the above articles as a source of the definition of dysbiosis. Not themselves however about microbiota and dysbiosis.

Coding: C = Change, I = Imbalance, S = Specific, O = Other.

Long definitions (quotes)	Reference	type
'a breakdown in the balance between putative species of "protective" versus "harmful" bacteria' (p. 1)	Tamboli et al., Gut 53:1-4, 2004.	I
A number of terms are used in the literature to describe either the state of balance" within the microbial population or the status of particular microbial groups... The balance... can be called "eubiosis". The opposite situation is termed "dysbiosis" (Haenel and Bendig 1975; Gedek 1975). Although not precisely defined this unstable state refers to qualitative and quantitative changes in the intestinal flora their metabolic activity and their local distribution. Metabolic activity and certain turnover rates may be more important than actual numbers of bacterial species. The situations "eubiosis" and "dysbiosis" may not be explained only by numbers of leading species and their definition therefore has limitations' (p. 87)."	Holzapfel et al., Int J Food Microbiol 41:85-101, 1998.	I
